# Proteomics-Guided Computational Prioritization of Putative RANKL-Binding Peptides from Proteins Identified in Deer Horn Glue

**DOI:** 10.3390/ijms27188255

**Published:** 2026-09-16

**Authors:** Zhonghao Fan, Tiefeng Sun, Heng Aik Teng, Haitao Du, Kun Yang, Cheng Wang, Jingwen Ma, Ping Wang

**Affiliations:** 1 School of Pharmacy, Shandong University of Traditional Chinese Medicine, Jinan 250355, China; fzh01225@163.com; 2 Institute of Chinese Medicine Pharmacology (Pharmacological Experimentation Center), Shandong Academy of Chinese Medicine, National Engineering Research Center for Modernization of Traditional Chinese Medicine Qilu Resources Branch, Shandong Center of Technology Innovation for Plant-Based Protein, Jinan 250014, China; suntiefenglove@163.com (T.S.); kkitdht@foxmail.com (H.D.); 18306390275@163.com (C.W.); 3 School of Traditional Chinese Medicine, INTI International University, Nilai 71800, Malaysia; aikteng.heng@newinti.edu.my; 4 The First Clinical Medical College, Shandong University of Traditional Chinese Medicine, Jinan 250355, China; kky0128@163.com; 5 Department of Science and Education, Shandong Academy of Chinese Medicine, National Engineering Research Center for Modernization of Traditional Chinese Medicine Qilu Resources Branch, Shandong Center of Technology Innovation for Plant-Based Protein, Jinan 250014, China; 6 Shandong Academy of Chinese Medicine, National Engineering Research Center for Modernization of Traditional Chinese Medicine Qilu Resources Branch, Shandong Center of Technology Innovation for Plant-Based Protein, Jinan 250014, China

**Keywords:** Deer Horn Glue, proteomics, bioactive peptides, RANKL, molecular dynamics, computational alanine scanning

## Abstract

Deer Horn Glue is rich in collagen- and tissue-derived proteins, but the link between its experimentally observed proteome, the theoretical peptide sequence space derived from that proteome, and bone-related molecular targets remains poorly defined. This study established a proteomics-guided multiscale computational workflow to prioritize putative receptor activator of nuclear factor-κB ligand (RANKL)-binding peptide candidates generated from proteins identified in one Deer Horn Glue sample. The Deer Horn Glue proteome was characterized by liquid chromatography–tandem mass spectrometry (LC-MS/MS). Experimentally identified proteins were subjected to in silico tryptic digestion and stepwise activity, safety, and physicochemical screening. All 49 retained candidates underwent global and interface-focused docking. Five prioritized peptides were examined by AlphaFold 3, three independently seeded 200 ns molecular dynamics simulations per complex, entropy-omitted molecular mechanics/generalized Born surface area (MM/GBSA) analysis, residue decomposition, and locally relaxed computational alanine substitution analysis. Proteomic analysis retained 707 target protein groups and generated 24,575 nonredundant theoretical peptide sequences. Stepwise screening retained 49 candidates. Global and interface-focused docking rankings showed modest agreement, and the expanded interface analysis identified additional candidates while retaining GASLQDWDFGK as the top-ranked sequence. Across three independent simulations, GASLQDWDFGK showed consistently low peptide root-mean-square deviation (RMSD), whereas HEFSVDMTCEGCSNAVTR formed the largest mean number of interfacial hydrogen bonds. Their entropy-omitted MM/GBSA estimates were generally the most favorable, although the order varied among simulations. Locally relaxed alanine substitutions highlighted reproducible energy-sensitive positions for experimental testing. By linking an experimentally observed Deer Horn Glue proteome to multiscale structural analysis, this workflow provides a traceable and reproducible strategy for prioritizing testable peptide–RANKL interaction hypotheses. The five candidates remain theoretical products of in silico digestion and require targeted detection, direct binding, and functional validation.

## 1. Introduction

Deer Horn Glue is a traditional animal-derived gelatinous medicinal material prepared from ossified cervid antlers through decoction, concentration, and drying processes [1]. Its major constituents are derived from collagen and other tissue proteins that undergo thermal denaturation during processing [1,2]. Beyond the evaluation of crude extracts, characterization of the protein composition and identification of functional peptides potentially released during processing or gastrointestinal digestion may provide a more precise understanding of its bioactive material basis [3,4]. Previous studies have reported that enzymatic hydrolysates derived from deer antler and Deer Horn Glue contain peptides with potential regulatory effects on bone metabolism [5]. For example, TSKYR and its fragments TSK and YR have been shown to promote osteoblast proliferation, mineralized nodule formation, and calcium uptake, accompanied by modulation of osteogenic markers such as runt-related transcription factor 2 (RUNX2) and osteocalcin (OCN) [3]. In addition, polypeptide and polysaccharide fractions derived from deer antler have demonstrated inhibitory effects on bone resorption in high-turnover bone loss models [6]. Together, these observations support Deer Horn Glue as a source of protein-derived peptide precursors. Yet, most studies have examined crude extracts, mixed hydrolysates, or isolated peptide candidates, leaving the experimentally observed parental proteome and its connection to target-oriented peptide discovery insufficiently defined [6,7].

Postmenopausal osteoporosis is a systemic skeletal disorder primarily associated with estrogen deficiency-induced disruption of bone remodeling homeostasis [8]. Reduced estrogen levels alter the bone marrow microenvironment and promote the production of inflammatory cytokines, including tumor necrosis factor-α, interleukin-1, and interleukin-6, thereby enhancing osteoclast differentiation and bone resorption [8,9]. Among the regulatory pathways involved, the receptor activator of nuclear factor-κB ligand (RANKL)-receptor activator of nuclear factor-κB (RANK)-osteoprotegerin (OPG) axis plays a central role in osteoclastogenesis. RANKL binding to RANK initiates osteoclast precursor differentiation, fusion, and maturation, whereas OPG functions as a soluble decoy receptor that competitively inhibits RANKL-mediated signaling [10,11]. The imbalance between RANKL and OPG caused by estrogen deficiency is therefore considered a major molecular mechanism contributing to excessive bone loss after menopause [8,10,11]. Although RANKL-targeting therapies have shown clinical efficacy in osteoporosis treatment [12,13], long-term application remains limited by adverse effects, treatment discontinuation-associated rebound bone turnover, and other clinical considerations [14,15,16]. These considerations make RANKL a rational molecular target for exploratory peptide screening. However, predicted peptide–RANKL interactions cannot establish inhibition of RANKL signaling or anti-osteoporotic efficacy, both of which require direct experimental validation [17,18].

Mass spectrometry-based proteomics provides a powerful approach for systematically characterizing protein components in complex biological materials and enables the tracing of candidate peptides back to their parental proteins [3,19]. In silico digestion based on experimentally identified proteins can further expand the peptide discovery space by predicting potential proteolytic products under defined cleavage rules [20,21]. Recently, integrated computational approaches combining bioactivity prediction, toxicity assessment, physicochemical evaluation, molecular docking, and molecular dynamics simulations have been increasingly applied to accelerate the identification of natural bioactive peptides [21,22,23,24].

In this study, we used liquid chromatography–tandem mass spectrometry (LC-MS/MS) to define the protein sequence space experimentally observed in Deer Horn Glue and generated a candidate library by in silico tryptic digestion of those identified proteins. The library was narrowed by sequential activity, safety, and physicochemical filters, followed by global hierarchical computational protein–peptide docking (HPEPDOCK) screening. Five prioritized peptides were then examined by interface-focused AutoDock Vina docking, AlphaFold 3 complex prediction, three independently seeded 200 ns molecular dynamics (MD) simulations, molecular mechanics/generalized Born surface area (MM/GBSA) analysis, residue decomposition, and locally relaxed computational alanine scanning. By linking experimentally detected parental proteins to progressively prioritized theoretical peptide candidates, the workflow was designed to provide a traceable basis for subsequent targeted detection and functional testing rather than to claim biological efficacy from computation alone.

## 2. Results

### 2.1. Proteomic Identification and Peptide Characteristics of Deer Horn Glue

**Figure 1:**
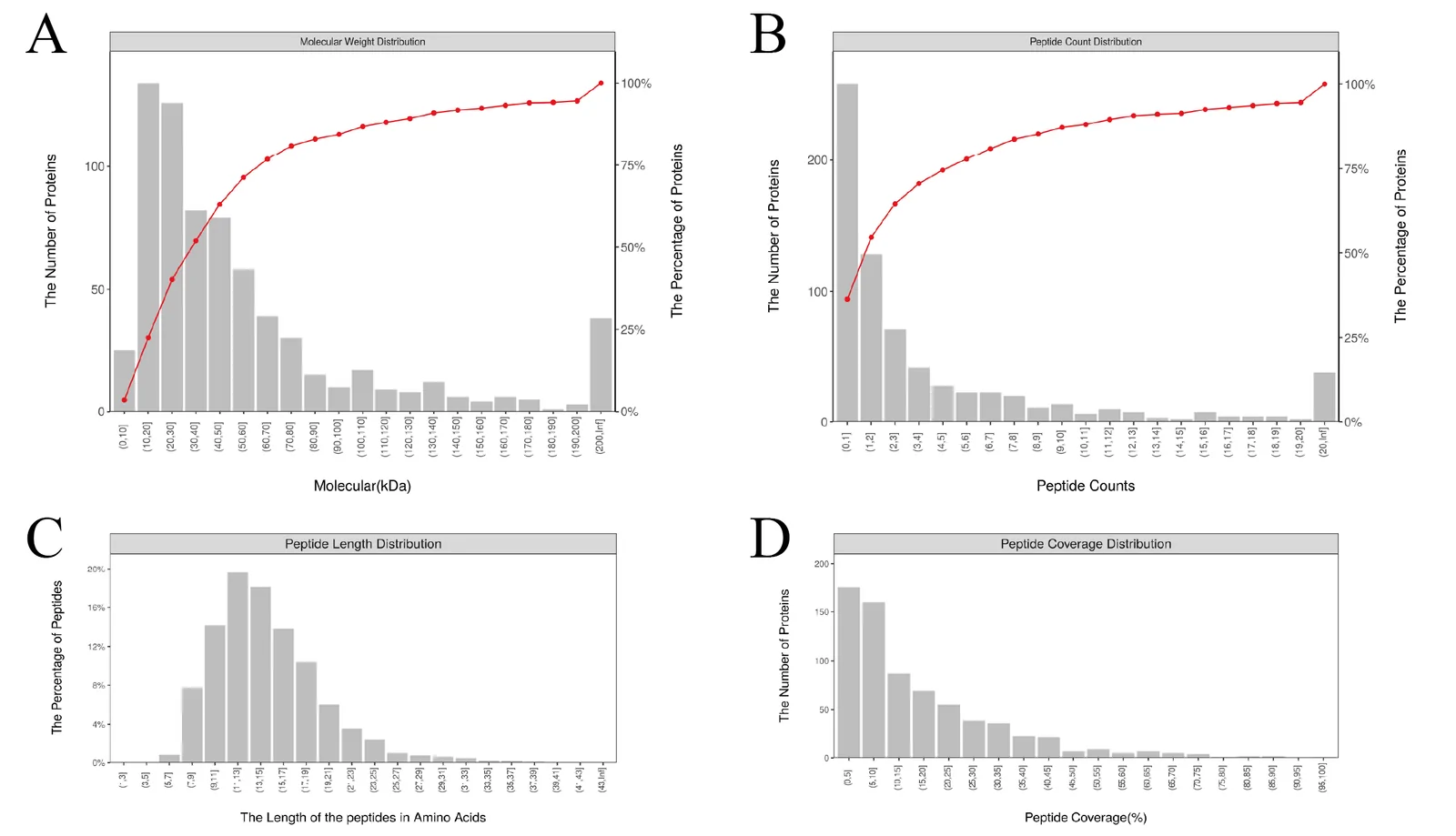
Proteomic identification characteristics of the analyzed Deer Horn Glue sample. (**A**) Molecular-mass distribution of the 707 retained target protein groups; (**B**) distribution of peptide counts assigned to the retained protein groups; (**C**) length distribution of the identified peptide sequences; and (**D**) sequence-coverage distribution of the protein groups. Bars show counts within each interval; the red curves in (**A**,**B**) show cumulative percentages.

The unfiltered MaxQuant proteinGroups table contained 768 entries. After reverse and potential-contaminant entries were excluded, 707 target protein groups were retained and used as the sequence resource for subsequent peptide mining. Fifteen groups had no unique peptide, 312 had one unique peptide, and 380 had at least two unique peptides according to the group-level counts. Median sequence coverage was 11.2%. A further 15 groups were flagged as identified only by site; excluding these would retain 692 groups with a median coverage of 11.4%. The theoretical library was constructed from the initial set of 707 target protein groups to preserve the predefined analysis workflow (Figure 1A–D). Extended identification-quality distributions and representative annotated MS/MS spectra are provided in Figures S1 and S2.

**Figure 2:**
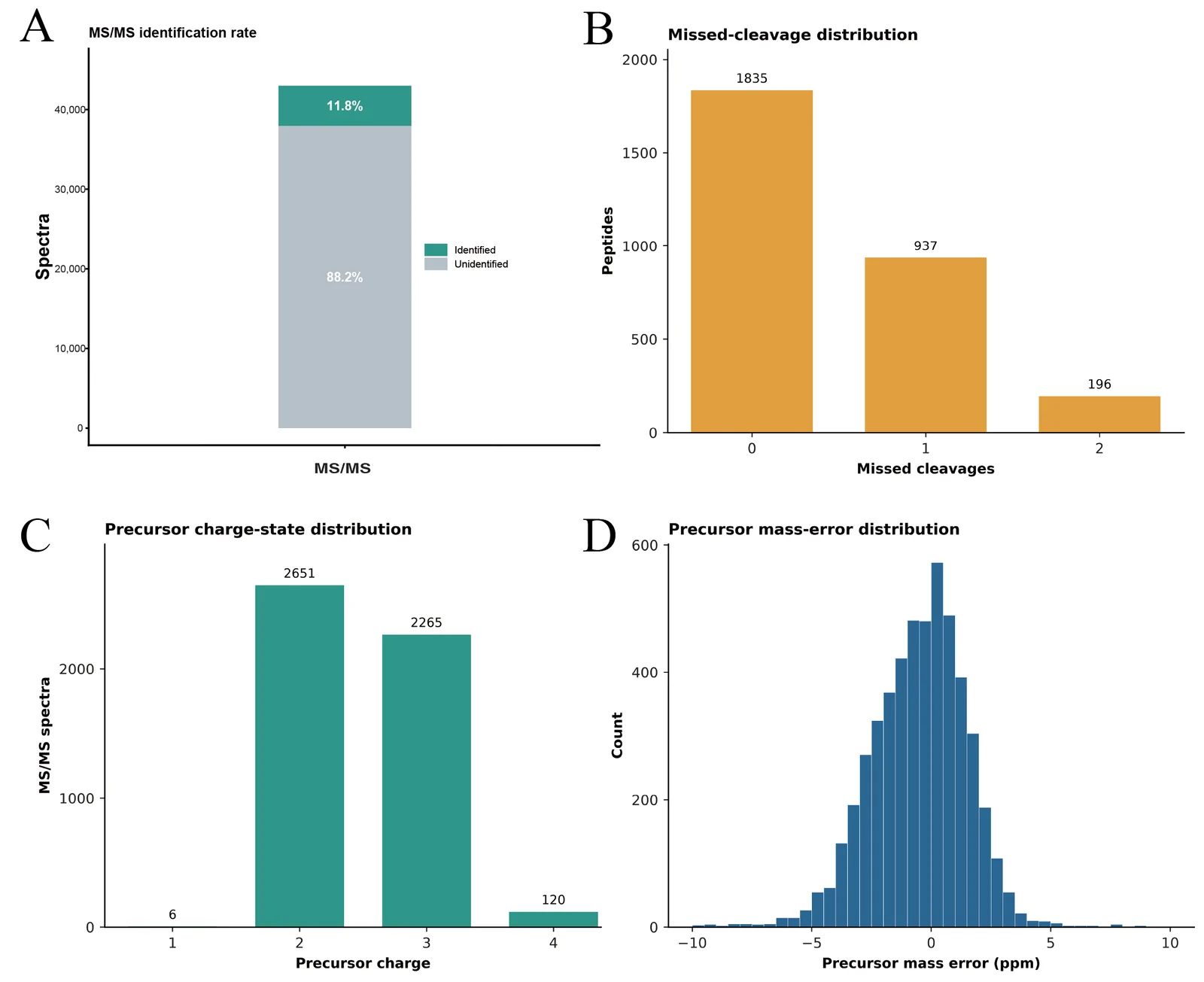
Tandem mass spectrometry (MS/MS) identification and precursor-ion characteristics of the analyzed Deer Horn Glue sample. (**A**) Identification outcome for 42,992 MS/MS queries submitted to MaxQuant, of which 5063 were identified (11.8%); (**B**) missed-cleavage distribution of the retained peptide sequences; (**C**) precursor charge-state distribution; and (**D**) precursor-ion mass-error distribution.

The MaxQuant summary recorded 42,992 submitted tandem mass spectrometry (MS/MS) queries and 5063 identified queries, corresponding to an identification fraction of 11.8% (Figure 2A). After reverse and potential-contaminant entries were removed, 2968 peptide sequences were retained. Peptides with zero, one, and two missed cleavages accounted for 1835, 937, and 196 sequences, respectively (Figure 2B). Precursor ions were mainly doubly and triply charged, accounting for 2651 and 2265 matched spectra (Figure 2C), and the precursor mass-error distribution was centered near 0 parts per million (ppm) (Figure 2D). Together, these metrics define the quality of the proteomic dataset used for subsequent in silico peptide generation.

**Figure 3:**
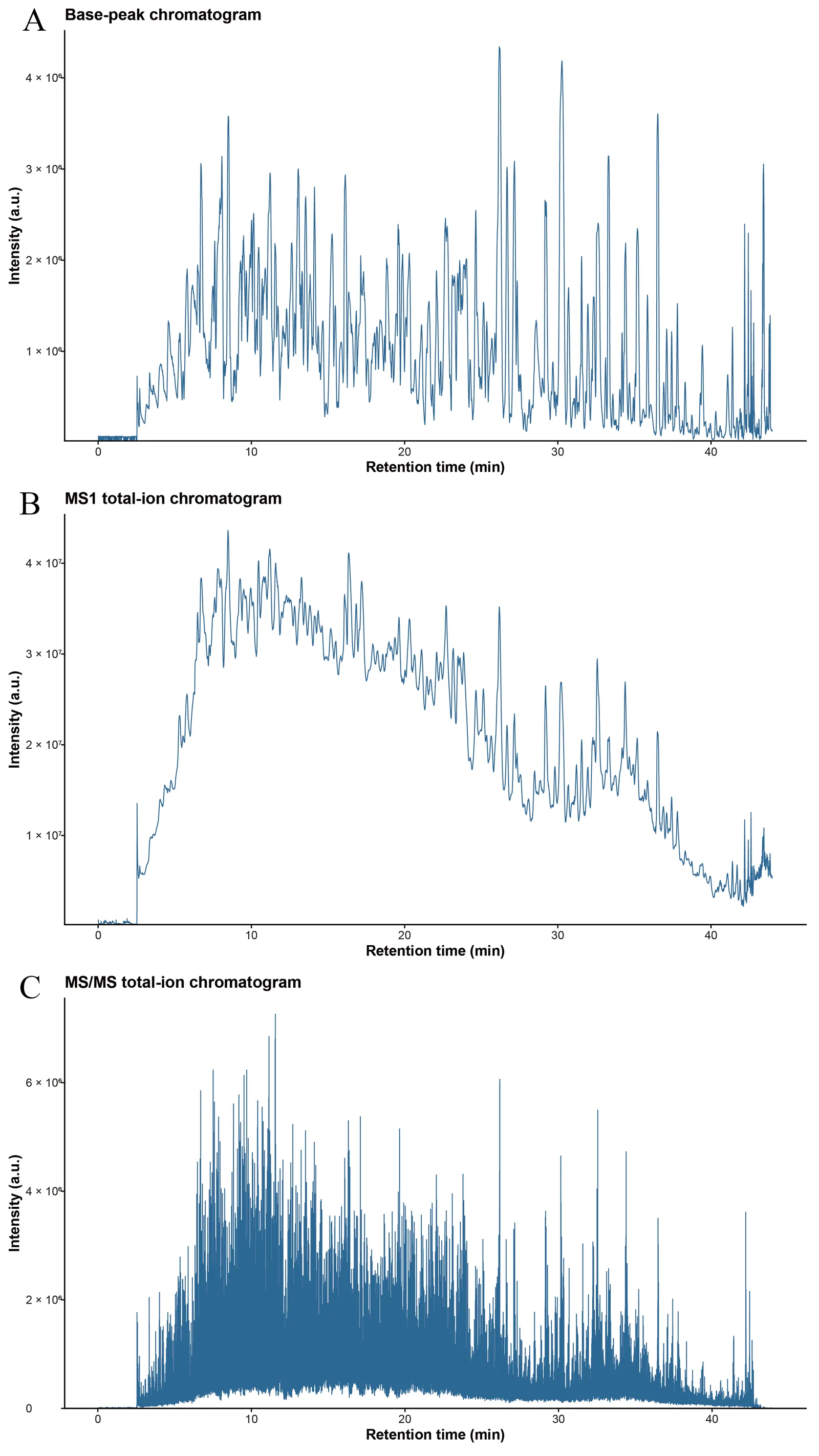
Liquid chromatography–tandem mass spectrometry (LC-MS/MS) chromatographic and ion-current characteristics of the analytical tryptic digest of the analyzed Deer Horn Glue sample. (**A**) Base-peak chromatogram; (**B**) first-stage mass spectrometry (MS1) total-ion chromatogram; and (**C**) tandem mass spectrometry (MS/MS) total-ion chromatogram. In all panels, the *x*-axis denotes retention time and the *y*-axis denotes ion-signal intensity in arbitrary units.

The base-peak chromatogram showed continuous peptide signals across the main separation period (approximately 5–43 min) (Figure 3A). MS1 total-ion current was relatively high during the early and middle retention-time regions and declined thereafter, while MS/MS ion current covered the principal peptide-elution window (Figure 3B,C). Together with the precursor mass-error and charge-state distributions, these chromatographic profiles provided quality-control context for the proteomic dataset used in subsequent in silico peptide generation.

### 2.2. Functional Annotation of Identified Proteins

**Figure 4:**
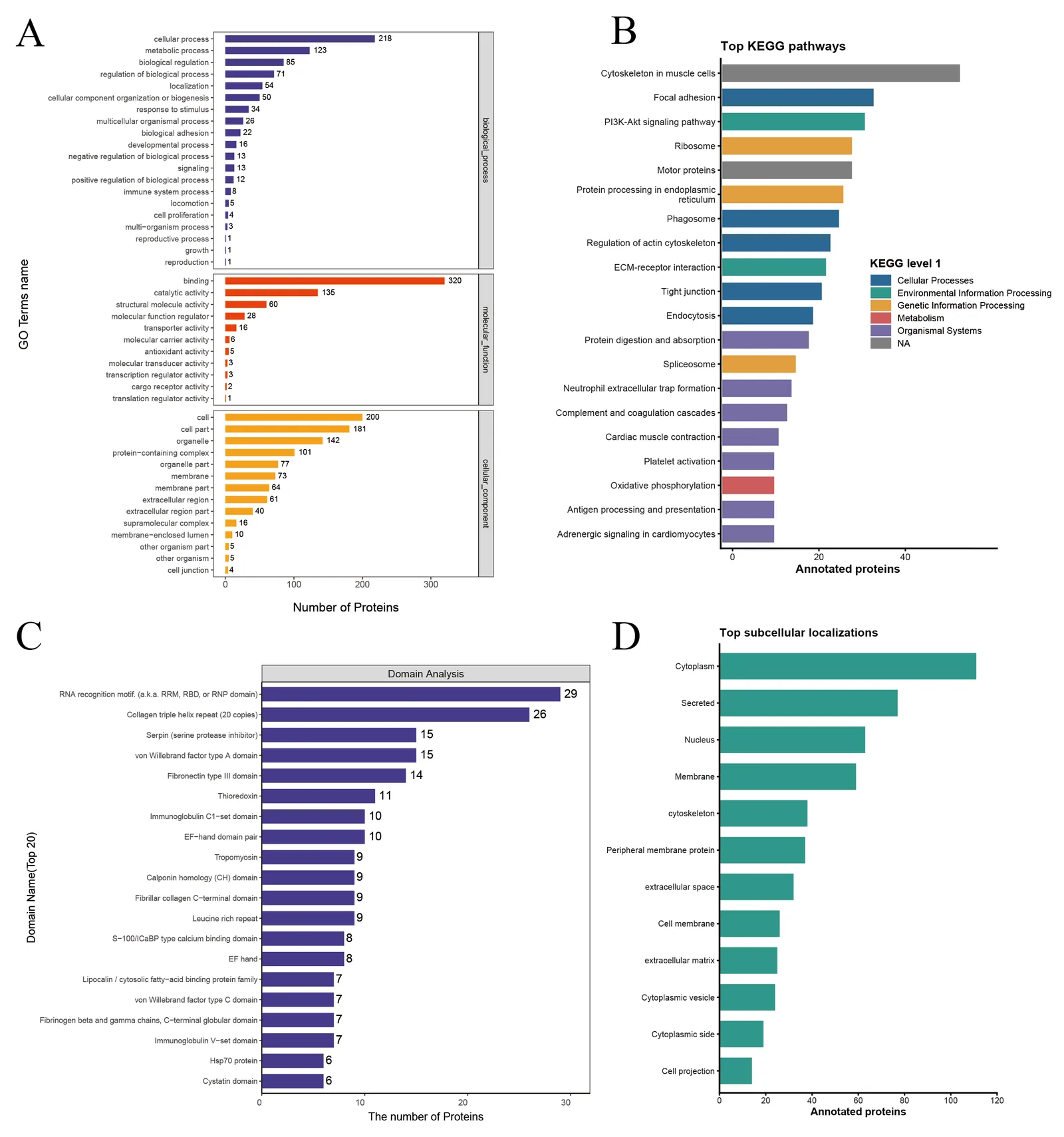
Functional annotation, domain composition, and predicted subcellular localization of protein groups identified in the analyzed Deer Horn Glue sample. (**A**) Gene Ontology (GO) level-2 classification across biological process, molecular function, and cellular component; (**B**) Kyoto Encyclopedia of Genes and Genomes (KEGG) pathway classification, with colors indicating KEGG level-1 categories; (**C**) the 20 most frequent annotated protein domains; and (**D**) predicted subcellular localization. These panels show annotation counts and category distributions, not statistical enrichment.

Functional annotation was performed for the 707 identified protein groups based on Gene Ontology (GO), Kyoto Encyclopedia of Genes and Genomes (KEGG), protein domains, and predicted subcellular localization analyses (Figure 4).

GO classification showed that the identified proteins were distributed among biological process, molecular function, and cellular component categories (Figure 4A). Within biological processes, cellular processes and metabolic processes represented the largest functional groups, containing 218 and 123 proteins, respectively. For molecular functions, binding activity was the most frequently annotated category, involving 320 proteins, followed by catalytic activity and structural molecule activity with 135 and 60 proteins, respectively. Regarding cellular components, proteins were mainly assigned to cell, cell part, and organelle categories.

KEGG annotation revealed that identified proteins were associated with multiple biological pathways, including cytoskeleton regulation in muscle cells, focal adhesion, and phosphoinositide 3-kinase (PI3K)–protein kinase B (Akt) signaling pathways (Figure 4B). Additional annotations were observed in ribosome function, protein processing in the endoplasmic reticulum, phagosome formation, regulation of actin cytoskeleton, extracellular matrix-receptor interaction, oxidative phosphorylation, and immune-related pathways.

Domain analysis showed that ribonucleic acid (RNA)-recognition-motif-related domains and collagen triple-helix repeat domains were among the most frequently detected protein domains, occurring in 29 and 26 proteins, respectively (Figure 4C). Other frequently observed domains included serine protease inhibitor domains, von Willebrand factor type A domains, and fibronectin type III domains. Predicted subcellular localization indicated that the identified proteins were mainly distributed in the cytoplasm, followed by secretory pathways, nucleus, and membrane-associated regions (Figure 4D).

### 2.3. In Silico Digestion and Stepwise Candidate-Peptide Screening

**Figure 5:**
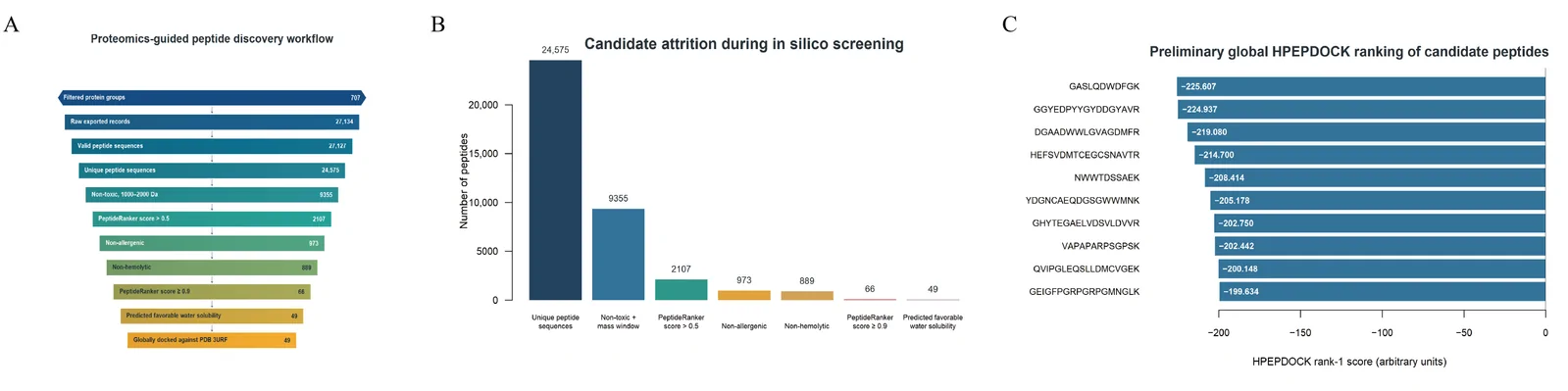
Stepwise computational prioritization of theoretical peptide candidates generated from proteins identified in the analyzed Deer Horn Glue sample. (**A**) Proteomics-guided peptide-discovery workflow and numbers retained at successive stages; (**B**) candidate attrition after in silico digestion and filtering by molecular mass, toxicity, predicted bioactivity, allergenicity, hemolytic potential, high-activity score, and water solubility; and (**C**) the 10 highest-ranked candidates according to the hierarchical computational protein–peptide docking (HPEPDOCK) rank-1 global-docking score. More negative HPEPDOCK scores indicate more favorable values under this scoring function and are used only for relative computational prioritization.

Database sequences associated with the 707 target protein groups were subjected to in silico digestion. The archived PeptideMass export contained 27,127 peptide records and seven non-sequence modification-annotation rows, which were excluded. Removal of embedded whitespace and exact-sequence deduplication yielded 24,575 unique theoretical sequences (Figure 5A).

Sequential filtering was performed according to molecular mass, toxicity prediction, bioactivity prediction, allergenicity, hemolytic potential, and water solubility. Among the generated peptides, 9355 sequences with molecular masses ranging from 1000 to 2000 Da were predicted to be non-toxic. PeptideRanker screening retained 2107 candidates with scores above 0.5. Subsequent AllerTOP v.2 analysis identified 973 non-allergenic candidates, of which 889 peptides further passed the hemolysis prediction filter. Applying a stricter PeptideRanker threshold (≥0.9) retained 66 high-scoring candidates, and PepCalc evaluation finally identified 49 peptides with favorable predicted water solubility for further RANKL-targeted structural analysis (Figure 5A,B).

Overall, sequential computational triage reduced the library from 24,575 theoretical sequences to 49 candidates for RANKL-focused structural analysis, providing an efficient bridge from proteomic identification to target-oriented peptide prioritization. The screening scores were used as prioritization metrics rather than experimental measures of activity or safety.

### 2.4. HPEPDOCK Global Docking Prioritizes Candidate RANKL-Binding Peptides

All 49 retained theoretical peptides were first subjected to global HPEPDOCK screening and were then evaluated using the same interface-focused AutoDock Vina protocol. Global and interface-focused ranks showed modest agreement (Spearman’s ρ = 0.420, *p* = 0.00265). Complete scores and ranks are provided in Table S1.

The five sequences initially selected by global HPEPDOCK ranking were GASLQDWDFGK, GGYEDPYYGYDDGYAVR, DGAADWWLGVAGDMFR, HEFSVDMTCEGCSNAVTR, and NWWTDSSAEK. Their interface-focused ranks among all 49 candidates were 1, 4, 19, 13, and 18, respectively. The interface-focused top five also included VCINSEHSVDTLLETLGK, YDGNCAEQDGSGWWMNK, and DGVGQEPVHLESPAQEHR, demonstrating that global and interface-focused docking provide complementary prioritization. The predicted properties and docking scores of these five peptides are summarized in Table 1.

The original five were retained as the predefined set for direct comparison across docking, AlphaFold 3, molecular dynamics, and MM/GBSA. The expanded 49-peptide interface analysis served as a sensitivity analysis and identified complementary candidates for future testing.

**Table 1:** Predicted properties and docking scores of the five prioritized putative RANKL-binding peptides. Abbreviations: RANKL, receptor activator of nuclear factor-κB ligand; RRM, RNA recognition motif; aa, amino acids; MW, molecular weight; Da, dalton; pI, isoelectric point; HPEPDOCK, hierarchical computational protein–peptide docking.

Sequence	GASLQDWDFGK	GGYEDPYYGYDDGYAVR	DGAADWWLGVAGDMFR	HEFSVDMTCEGCSNAVTR	NWWTDSSAEK
Parent protein	Tankyrase 1-binding protein C-terminal domain-containing protein	RRM domain-containing protein	HN1L	Copper transport protein ATOX1	Fibrillar collagen NC1 domain-containing protein
UniProt accession	A0A212DIY6	A0A5J5MMQ1	A0A212CYR2	A0A212D0L8	A0A5J5MKI9
Position in parent protein	921–931	484–500	17–32	4–21	1265–1274
Length (aa)	11	17	16	18	10
MW (Da)	1223.29	1959.97	1766.93	1986.18	1223.25
pI	3.71	3.32	3.41	4.31	3.93
Net charge at pH 7.0	−1	−3	−2	−2	−1
PeptideRanker score	0.976	0.950	0.946	0.987	0.977
Toxicity (ToxinPred)	Non-toxic	Non-toxic	Non-toxic	Non-toxic	Non-toxic
Allergenicity (AllerTOP v.2)	Non-allergenic	Non-allergenic	Non-allergenic	Non-allergenic	Non-allergenic
Hemolysis (HemoPI-2)	Non-hemolytic	Non-hemolytic	Non-hemolytic	Non-hemolytic	Non-hemolytic
Water solubility (PepCalc)	Good	Good	Good	Good	Good
HPEPDOCK rank-1 score	−225.607	−224.937	−219.080	−214.700	−208.414
Vina score (kcal/mol)	−12.070	−11.291	−10.438	−10.751	−10.502
MaxQuant protein-group ID	408	445	261	277	433
Associated accessions	A0A212DIY6; A0A5N3XAW3	A0A5J5MMQ1	A0A212CYR2; A0A5J5MRX8	A0A212D0L8; A0A5J5N476	A0A5J5MKI9
Group-level unique peptides	3	1	3	2	3
Sequence coverage (%)	3.9	2.1	22.7	52.9	9.1
Candidate sequence matched in analytical tryptic digest	No	No	No	Yes; sequence-matched assignment	No

### 2.5. AutoDock Vina Redocking and Interfacial Binding Modes

**Figure 6:**
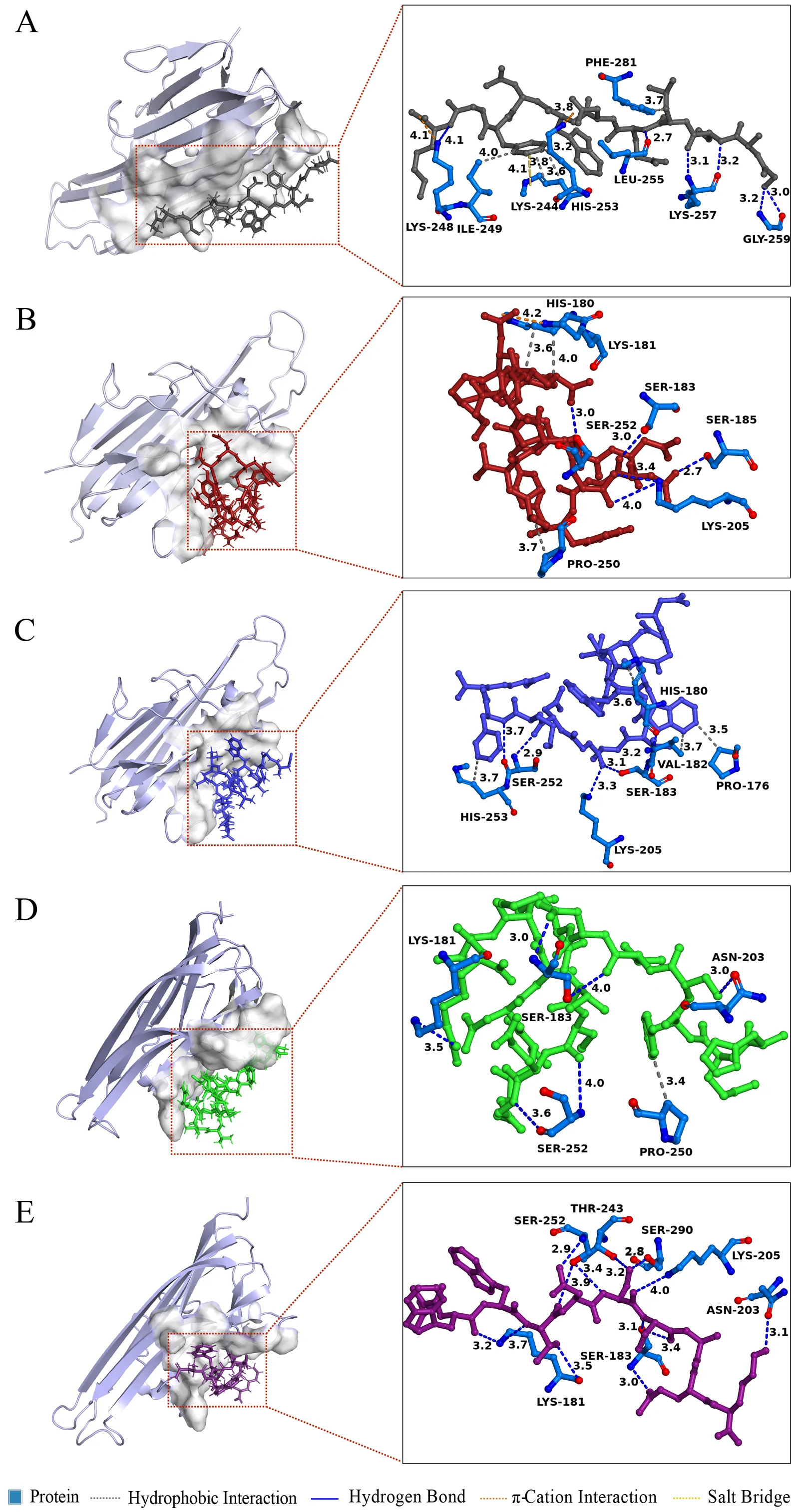
Representative AutoDock Vina pose hypotheses and interfacial contacts for the five initially prioritized theoretical peptide–RANKL systems. (**A**) GASLQDWDFGK-RANKL; (**B**) GGYEDPYYGYDDGYAVR-RANKL; (**C**) DGAADWWLGVAGDMFR-RANKL; (**D**) HEFSVDMTCEGCSNAVTR-RANKL; and (**E**) NWWTDSSAEK-RANKL. In each panel, the left view shows the overall pose on the RANKL surface and the right view enlarges the local interface. Dashed lines and numeric labels indicate modeled interfacial interactions and the corresponding interatomic distances (Å), including hydrogen-bond, hydrophobic interactions, salt bridges, and a π-cation interaction. RANKL residue numbering follows Protein Data Bank (PDB) structure 3URF. The rigid-docking models are starting-pose hypotheses rather than experimental binding structures.

Interface-focused Vina docking produced poses for all 49 candidates within a search box centered on the RANKL-OPG interface. Figure 6 presents the five initially prioritized systems as representative pose hypotheses. The rigid-receptor calculations were used to define starting structures for molecular dynamics simulation. The starting conformers and representative AutoDock Vina poses are detailed in Table S2.

For the five initially prioritized candidates, Vina scores ranged from −12.070 to −10.438 kcal/mol. GASLQDWDFGK had the lowest score within this subset and ranked first among all 49 candidates in the expanded interface-focused analysis. These scoring values were used to select pose hypotheses for simulation.

The displayed poses contacted residues within the predefined RANKL interface, including Lys181, Lys205, Lys244, Lys248, Ser251, His253, Lys257, Lys282, and Arg284. These recurrent contacts define structure-based hypotheses for subsequent experimental evaluation.

Matched docking controls were evaluated using the same two-stage workflow. HPEPDOCK scores were more negative for the five candidates than for the 15 composition-preserving scrambled controls (U = 13.0, one-sided *p* = 0.0164), whereas Vina scores were not significantly different (U = 36.0, *p* = 0.4664). Parent-matched analyses produced the same interpretation for HPEPDOCK (*p* = 0.0313) and Vina (*p* = 0.7813). YR-11 yielded HPEPDOCK and Vina scores of −195.600 and −11.463 kcal/mol, respectively; as a single literature reference, it was interpreted descriptively rather than as a positive-control distribution.

### 2.6. AlphaFold 3 Confidence Assessment of Candidate Peptide–RANKL Models

**Figure 7:**
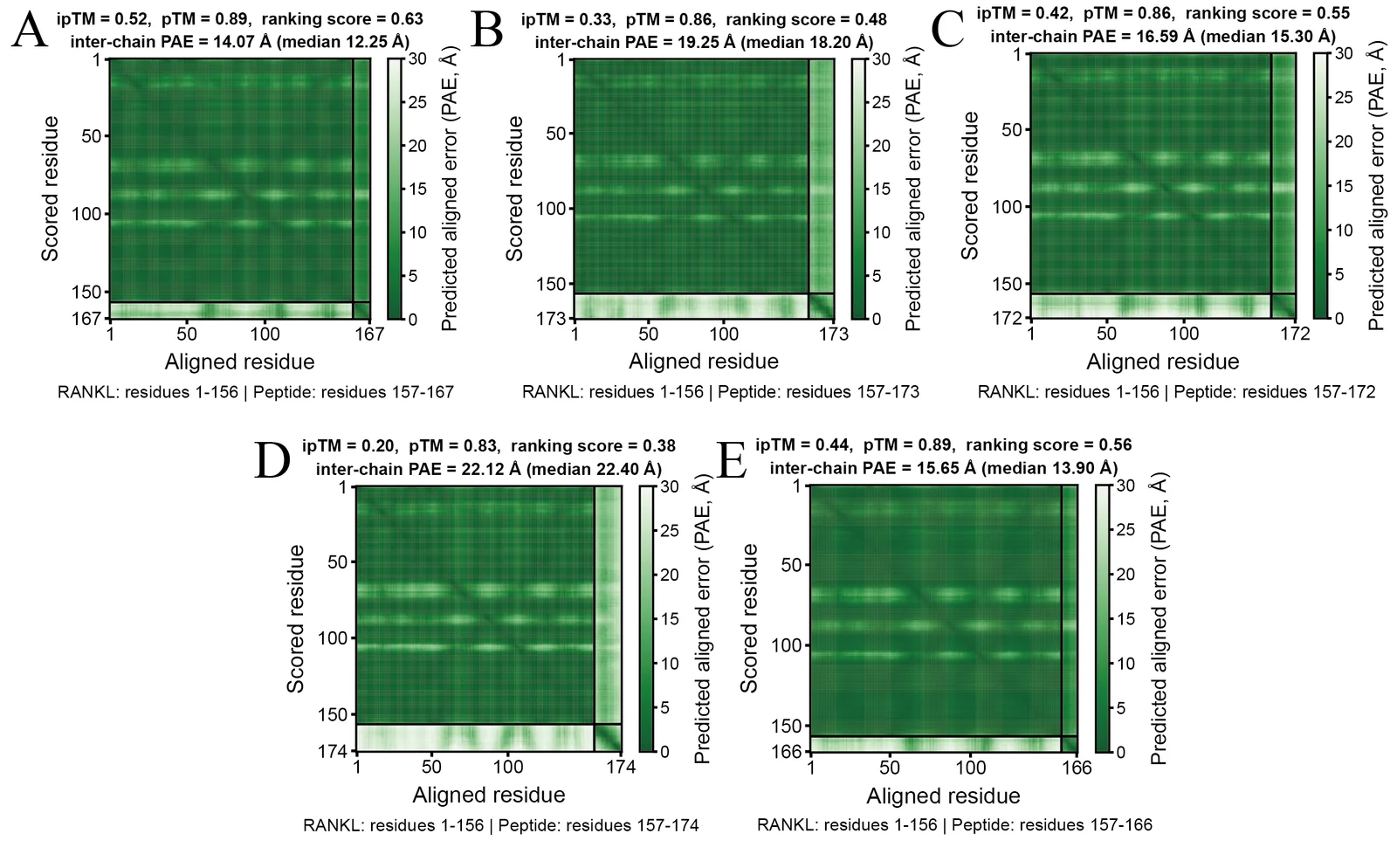
AlphaFold 3 predicted-aligned-error matrices and confidence metrics for the five candidate peptide–RANKL models. (**A**) GASLQDWDFGK-RANKL: ipTM = 0.52, pTM = 0.89, ranking score = 0.63, mean interchain PAE = 14.07 Å, and median interchain PAE = 12.25 Å; (**B**) GGYEDPYYGYDDGYAVR-RANKL: ipTM = 0.33, pTM = 0.86, ranking score = 0.48, mean interchain PAE = 19.20 Å, and median interchain PAE = 18.20 Å; (**C**) DGAADWWLGVAGDMFR-RANKL: ipTM = 0.42, pTM = 0.86, ranking score = 0.55, mean interchain PAE = 16.60 Å, and median interchain PAE = 15.30 Å; (**D**) HEFSVDMTCEGCSNAVTR-RANKL: ipTM = 0.20, pTM = 0.83, ranking score = 0.38, mean interchain PAE = 22.10 Å, and median interchain PAE = 22.40 Å; and (**E**) NWWTDSSAEK-RANKL: ipTM = 0.44, pTM = 0.89, ranking score = 0.56, mean interchain PAE = 15.60 Å, and median interchain PAE = 13.90 Å. The axes denote residue positions, and black boundaries separate the RANKL and peptide chains. Lower PAE denotes greater predicted confidence in relative residue placement. The low-to-moderate ipTM values and interchain PAE values indicate appreciable interface uncertainty; these models are confidence assessments rather than validation of binding. Abbreviations: RANKL, receptor activator of nuclear factor-κB ligand; ipTM, interface predicted template modeling score; pTM, predicted template modeling score; PAE, predicted aligned error.

AlphaFold 3 was used as an additional structure-prediction method for the five candidate peptide–RANKL complexes (Figure 7, Table 2). Confidence in the RANKL chain was high, whereas the peptide interfaces showed lower confidence. Extended interface-confidence metrics are provided in Table S3.

For GASLQDWDFGK-RANKL, the predicted interface predicted template modeling score (ipTM), predicted template modeling score (pTM), and ranking score were 0.52, 0.89, and 0.63, respectively. The corresponding values were 0.33, 0.86, and 0.48 for GGYEDPYYGYDDGYAVR; 0.42, 0.86, and 0.55 for DGAADWWLGVAGDMFR; 0.20, 0.83, and 0.38 for HEFSVDMTCEGCSNAVTR; and 0.44, 0.89, and 0.56 for NWWTDSSAEK.

Mean and median bidirectional interchain predicted aligned error (PAE) values were emphasized over the minimum value. Mean interchain PAE ranged from 14.07 Å for GASLQDWDFGK to 22.10 Å for HEFSVDMTCEGCSNAVTR, while median values ranged from 12.25 to 22.40 Å (Table 2), indicating appreciable uncertainty in peptide placement.

GASLQDWDFGK had the highest ipTM (0.52) and the lowest mean interchain PAE (14.07 Å) among the five systems, supporting its relative priority while also indicating that the predicted interface remains uncertain. Accordingly, AlphaFold 3 metrics were used to assess model confidence rather than as independent evidence of binding.

### 2.7. 200 ns Molecular Dynamics Simulations Reveal Distinct Complex Dynamics

**Figure 8:**
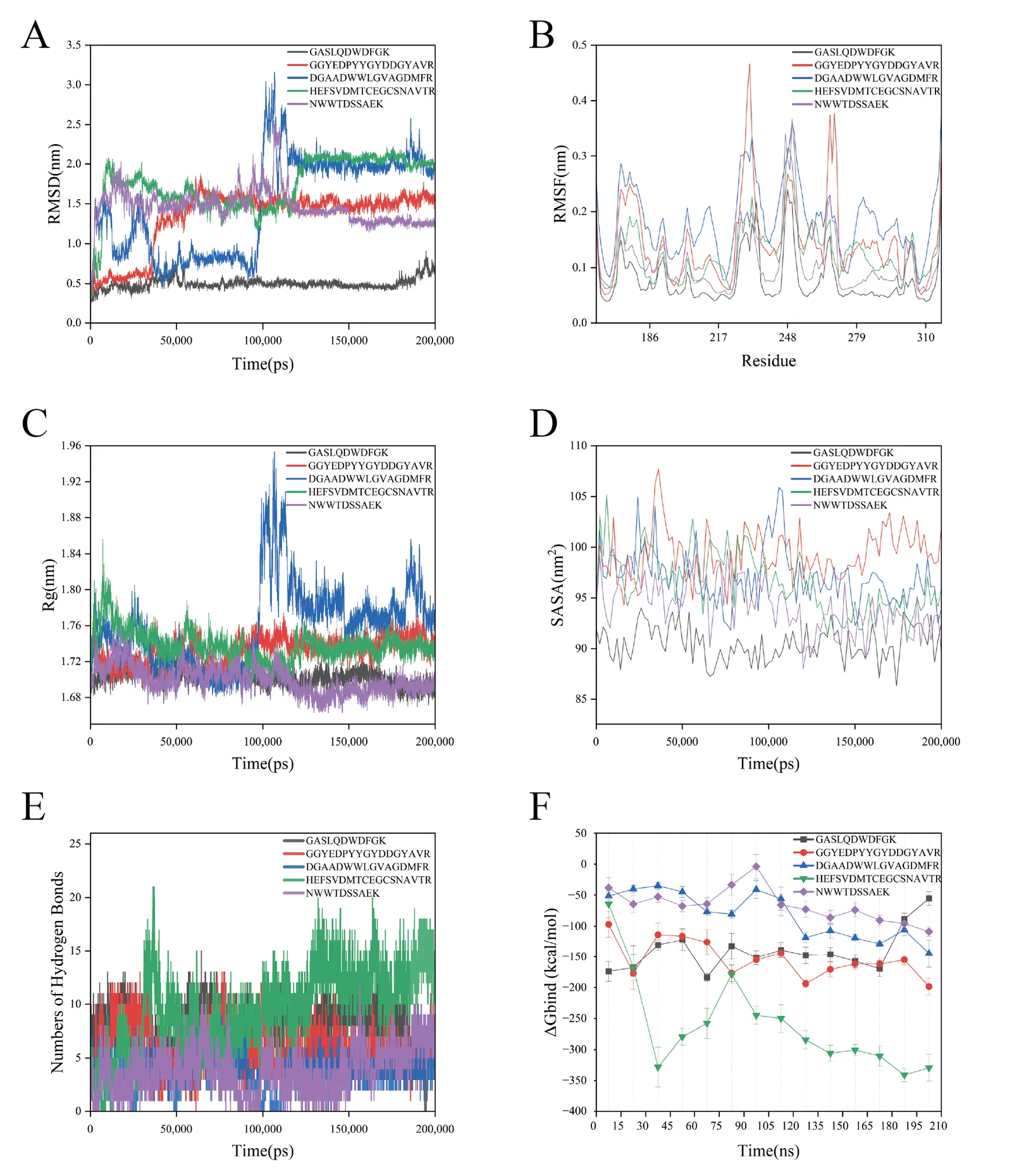
Representative 200 ns molecular-dynamics trajectories and temporal MM/GBSA profiles for the five candidate peptide–RANKL systems. (**A**) Peptide root-mean-square (RMSD) deviation after fitting each trajectory to the RANKL backbone; (**B**) root-mean-square fluctuation (RMSF) of RANKL residues; (**C**) radius of gyration (Rg) of the complex; (**D**) solvent-accessible surface area (SASA) of the complex; (**E**) number of peptide–RANKL hydrogen bonds; and (**F**) entropy-omitted molecular mechanics/generalized Born surface area (MM/GBSA) binding-energy estimates summarized over consecutive 15 ns windows. Curves show one representative independently seeded simulation per complex; the additional independent simulations and across-simulation summaries are provided in the Supplementary Materials. Variation among time points or frames describes correlated temporal fluctuations and is not an independent estimate of binding-affinity uncertainty.

Each peptide–RANKL system was analyzed in three independently seeded 200 ns molecular dynamics simulations, giving 15 production trajectories in total. The independent simulation, rather than individual time frames, was the replicate unit. Figure 8 shows one representative simulation; complete results from all independent simulations are provided in Figures S3–S6 and Tables S6–S8. The dynamic characteristics of the five complexes are summarized in Table 3.

Across the three independent simulations, mean peptide root-mean-square deviation (RMSD) values were 0.582 ± 0.103, 0.964 ± 0.319, 1.500 ± 0.489, 1.155 ± 0.511, and 1.468 ± 0.781 nm for GASLQDWDFGK, GGYEDPYYGYDDGYAVR, DGAADWWLGVAGDMFR, HEFSVDMTCEGCSNAVTR, and NWWTDSSAEK, respectively (mean ± sample standard deviation [SD] of three simulation means). The between-simulation variation demonstrates the sampling dependence of the peptide dynamics.

RANKL backbone fluctuations remained mainly localized to loop regions, whereas complex radius of gyration and solvent-accessible surface area showed system- and simulation-dependent variation. Across-simulation summaries for the complete trajectories and final 150–200 ns intervals are provided in Table S7.

Across the three independent simulations, HEFSVDMTCEGCSNAVTR formed the largest mean number of peptide–RANKL hydrogen bonds (9.864 ± 1.363), followed by GASLQDWDFGK (8.143 ± 0.161), GGYEDPYYGYDDGYAVR (5.990 ± 0.427), NWWTDSSAEK (4.112 ± 1.456), and DGAADWWLGVAGDMFR (3.373 ± 1.173). Values are mean ± sample SD of three simulation means.

GASLQDWDFGK showed the most reproducibly low peptide RMSD, whereas HEFSVDMTCEGCSNAVTR formed the largest mean number of interfacial hydrogen bonds. These complementary dynamic features supported their priority for further evaluation, while remaining descriptors of the sampled starting-pose dynamics rather than direct measures of affinity.

**Table 3:** Dynamic characteristics of the peptide–RANKL complexes during the 200 ns molecular-dynamics simulations. Values are the mean ± sample SD of three independently seeded trajectory means (*n* = 3), calculated over 0–200 ns. Abbreviations: RANKL, receptor activator of nuclear factor-κB ligand; RMSD, root-mean-square deviation; SD, standard deviation; H-bonds, hydrogen bonds.

Peptide	Peptide RMSD (nm)	Interfacial H-Bonds
GASLQDWDFGK	0.582 ± 0.103	8.143 ± 0.161
GGYEDPYYGYDDGYAVR	0.964 ± 0.319	5.990 ± 0.427
DGAADWWLGVAGDMFR	1.500 ± 0.489	3.373 ± 1.173
HEFSVDMTCEGCSNAVTR	1.155 ± 0.511	9.864 ± 1.363
NWWTDSSAEK	1.468 ± 0.781	4.112 ± 1.456

### 2.8. MM/GBSA Reveals Distinct Binding-Energy Profiles Among Candidate Peptides

Entropy-omitted MM/GBSA endpoint estimates were calculated from 101 snapshots from each of the three independently seeded 200 ns simulations per peptide. The three simulation means, rather than 303 correlated frames, were used to summarize uncertainty (Table 4 and Table S8).

Across-simulation estimates were −177.70 ± 31.61, −86.90 ± 57.96, −43.06 ± 40.37, −205.14 ± 54.43, and −50.55 ± 43.32 kcal/mol for GASLQDWDFGK, GGYEDPYYGYDDGYAVR, DGAADWWLGVAGDMFR, HEFSVDMTCEGCSNAVTR, and NWWTDSSAEK, respectively (mean ± sample SD of three simulation means). HEFSVDMTCEGCSNAVTR and GASLQDWDFGK generally yielded the most favorable values, but their ordering changed among simulations.

The estimates include van der Waals, electrostatic, polar generalized-Born, and nonpolar surface-area terms but omit conformational entropy. Differences in peptide length and net charge can also affect raw interaction and solvation terms. Consequently, these model-dependent values were used only for qualitative prioritization.

Time-resolved profiles showed substantial within- and between-simulation variation (Figure 8F and Figures S4–S6). Framewise variability describes temporal fluctuation among correlated conformations and was not used as an independent estimate of affinity uncertainty. Accordingly, the MM/GBSA results support qualitative prioritization rather than a definitive affinity ranking. The 15-ns window-resolved estimates for R1–R3 are provided in Table S4.

### 2.9. RANKL Residue Energy Decomposition Identifies Key Interfacial Sites

**Figure 9:**
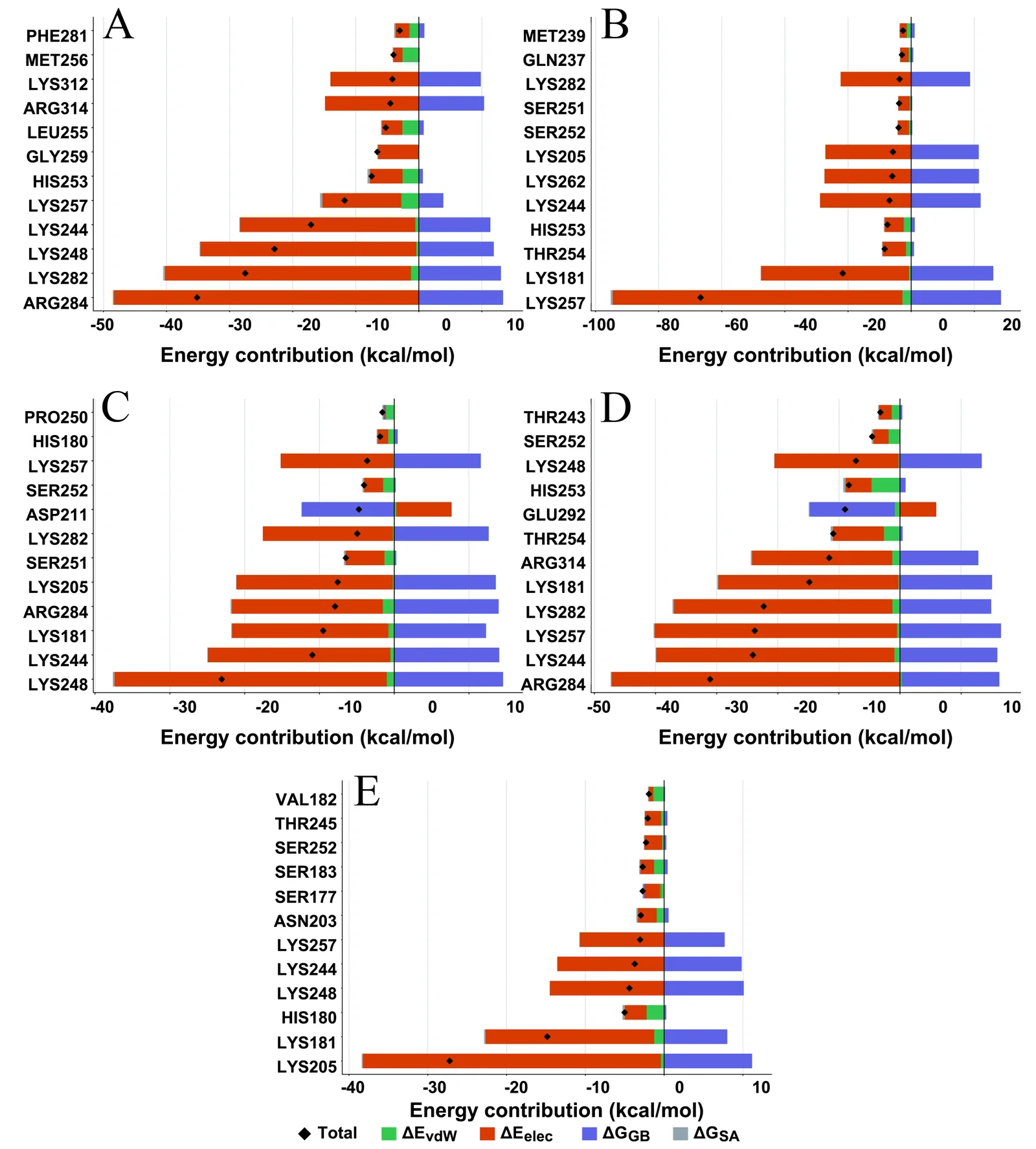
Representative MM/GBSA decomposition of modeled RANKL residue contributions in the five candidate peptide–RANKL systems. (**A**) GASLQDWDFGK-RANKL; (**B**) GGYEDPYYGYDDGYAVR-RANKL; (**C**) DGAADWWLGVAGDMFR-RANKL; (**D**) HEFSVDMTCEGCSNAVTR-RANKL; and (**E**) NWWTDSSAEK-RANKL. Bars show van der Waals (ΔEvdW), electrostatic (ΔEelec), polar-solvation (ΔGGB), and nonpolar-solvation (ΔGSA) terms; black diamonds show the total modeled contribution. Negative values indicate favorable contributions under the entropy-omitted MM/GBSA model. Energies are in kcal mol−1, and RANKL residue numbering follows PDB 3URF. The panels show one representative independently seeded simulation; decomposition results from the other independent simulations are supplied in the Supplementary Materials and are interpreted as sampling-sensitive hypotheses. Abbreviations: MM/GBSA, molecular mechanics/generalized Born surface area; RANKL, receptor activator of nuclear factor-κB ligand; PDB, Protein Data Bank.

Residue decomposition was performed separately for each independent simulation. Figure 9 and Table 5 present the representative replicate 1 (R1) results, whereas the corresponding replicate 2 (R2) and replicate 3 (R3) results are provided in Figures S7 and S8, respectively. Differences among simulations were interpreted as sampling sensitivity rather than as a consensus residue ranking.

For the GASLQDWDFGK-RANKL complex, Lys282, Lys244, Lys248, Arg284, and Lys257 showed the most favorable contributions, with energy contributions of −50.75, −50.03, −42.90, −33.06, and −26.81 kcal/mol, respectively (Figure 9A). In the GGYEDPYYGYDDGYAVR complex, Lys181 showed the strongest contribution (−53.27 kcal/mol), followed by Ser251, Lys257, Lys244, and Lys205 (Figure 9B).

For DGAADWWLGVAGDMFR-RANKL, Lys244, Lys282, Lys257, Arg284, and Lys262 contributed −56.68, −56.25, −53.86, −44.25, and −37.87 kcal/mol, respectively (Figure 9C). In the HEFSVDMTCEGCSNAVTR complex, Lys257 and Lys181 showed prominent contributions (−63.74 and −58.06 kcal/mol), while Lys205, Ser251, and Lys244 also contributed favorably (Figure 9D).

Compared with the other systems, residue contributions in the NWWTDSSAEK complex were relatively moderate. Lys181, Pro176, Lys205, Ile175, and Ser177 were the top contributing residues, with values of −15.18, −14.50, −14.25, −13.81, and −12.57 kcal/mol, respectively (Figure 9E).

Several charged RANKL residues appeared favorable in the representative analysis, but their magnitude and ordering varied among independent simulations. The recurrent residues therefore represent testable model-based contact hypotheses.

### 2.10. Locally Relaxed Computational Alanine Substitution Analysis

**Figure 10:**
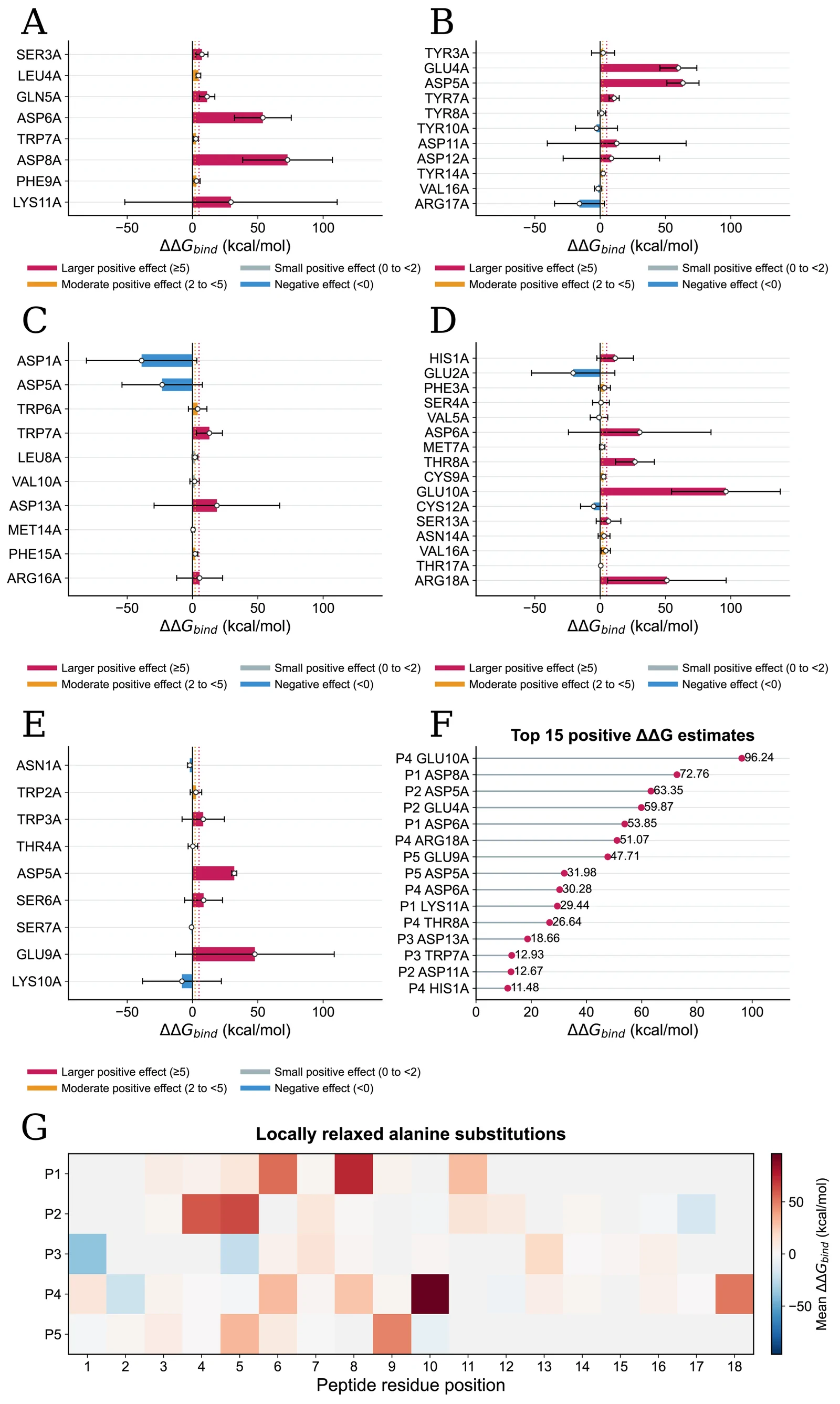
Locally relaxed computational alanine-substitution analysis of the five candidate peptides. (**A**) GASLQDWDFGK; (**B**) GGYEDPYYGYDDGYAVR; (**C**) DGAADWWLGVAGDMFR; (**D**) HEFSVDMTCEGCSNAVTR; and (**E**) NWWTDSSAEK. Panels (**A**–**E**) show ΔΔGbind = ΔGbind, mutant − ΔGbind, wild type for each scannable residue after independent minimization of the wild-type and standard alanine-mutant structures using the Onufriev–Bashford–Case II (OBC-II) implicit-solvent model. Positive values indicate weaker modeled binding after substitution, whereas negative values indicate a more favorable endpoint estimate after substitution. Colors denote large positive effects (≥5 kcal mol^−1^), moderate positive effects (2 to <5 kcal mol^−1^), small positive effects (0 to <2 kcal mol^−1^), and negative effects (<0 kcal mol^−1^). Error bars represent the sample standard deviation (SD) of three estimates obtained from independently derived 150–200 ns structural medoids, not the standard error of the mean (SEM) of trajectory frames. (**F**) The 15 largest positive across-simulation mean ΔΔGbind estimates among all five peptides. (**G**) Heatmap of the across-simulation mean ΔΔGbind by peptide sequence position; blank cells indicate residues not subjected to alanine substitution. No mutant-specific production simulation or conformational-entropy calculation was performed; therefore, the values are exploratory, model-dependent sensitivity estimates rather than experimental mutation free energies. Abbreviations: ΔGbind, binding free energy; ΔΔGbind, difference in binding free energy; WT, wild type.

A locally relaxed alanine substitution analysis was performed using one structural medoid selected from the 150–200 ns interval of each independent replicate simulation. Wild-type and standard AMBER ff14SB alanine-mutant structures were minimized independently using the Onufriev–Bashford–Case II (OBC-II) implicit-solvent model before endpoint energy differences were calculated. Complete results for 54 mutable positions are provided in Table S5. The alanine-substitution profiles are shown in Figure 10.

The three independently derived medoids showed marked between-simulation variability for several charged positions. The analysis therefore emphasized positions with consistently positive effects across all three medoids. Substitutions meeting the predefined descriptive criterion are summarized in Table 6.

For GASLQDWDFGK, S3A, Q5A, D6A, and D8A were positive in all three independently derived medoids and had mean estimates of 7.19, 11.14, 53.85, and 72.76 kcal/mol, respectively. K11A varied from −63.48 to 87.07 kcal/mol, demonstrating strong conformational sensitivity.

For GGYEDPYYGYDDGYAVR, E4A, D5A, and Y7A were consistently positive, with means of 59.87, 63.35, and 10.70 kcal/mol. R17A, which was unusually large in the original unrelaxed calculation, was not reproducibly positive after local relaxation.

For DGAADWWLGVAGDMFR, only W7A met the predefined descriptive criterion of a positive value in all three medoids with an across-simulation mean of at least 5 kcal/mol. Several other substitutions changed sign among simulations.

For HEFSVDMTCEGCSNAVTR, T8A, E10A, and R18A were consistently positive, with means of 26.64, 96.24, and 51.07 kcal/mol. For NWWTDSSAEK, D5A was consistently positive with a mean of 31.98 kcal/mol.

Large across-simulation standard deviations remained for several substitutions, including charged residues. The consistently positive positions identify energy-sensitive side chains under the local-minimization model and provide focused candidates for experimental mutagenesis.

The heatmap summarizes across-simulation means, while error bars show the sample SD of three independently derived medoid estimates. No mutant-specific production simulations or conformational-entropy calculations were performed; the results are therefore exploratory and intended to guide experimental mutagenesis.

**Table 6:** Locally relaxed alanine substitutions yielding positive ΔΔGbind estimates in all three medoids and an across-simulation mean ΔΔGbind ≥ 5 kcal/mol. Abbreviations: ΔΔGbind, difference in binding free energy; SD, standard deviation.

Peptide	Substitutions Meeting the Criterion, Mean ΔΔGbind ± Sample SD (kcal/mol)
GASLQDWDFGK	S3A, 7.19 ± 4.62; Q5A, 11.14 ± 5.93; D6A, 53.85 ± 21.74; D8A, 72.76 ± 34.31
GGYEDPYYGYDDGYAVR	E4A, 59.87 ± 14.12; D5A, 63.35 ± 12.32; Y7A, 10.70 ± 3.87
DGAADWWLGVAGDMFR	W7A, 12.93 ± 9.99
HEFSVDMTCEGCSNAVTR	T8A, 26.64 ± 14.88; E10A, 96.24 ± 41.59; S13A, 6.42 ± 9.42; R18A, 51.07 ± 45.34
NWWTDSSAEK	D5A, 31.98 ± 1.94

## 3. Discussion

Deer Horn Glue is an animal-derived gelatinous medicinal material mainly composed of collagen and other tissue-derived proteins. During processing, thermal treatment can disrupt the higher-order structure of collagen and induce partial cleavage or degradation of protein chains, leading to the generation of lower-molecular-weight peptide fractions [25,26]. Therefore, the potential biological basis of Deer Horn Glue should not be considered solely from the perspective of intact proteins or crude extracts; peptides released during processing and digestion may also contribute to its biological properties [3,26]. Previous studies have identified peptides derived from Deer Horn Glue and deer antler-related materials with potential regulatory effects on bone metabolism, including promotion of osteoblast mineralization, regulation of osteogenic marker expression, and inhibition of osteoclast-related responses [3,5]. However, most previous studies have focused on individual peptide identification or functional characterization of crude peptide mixtures, whereas the relationship between candidate peptides, their parental proteins, and potential molecular targets remains insufficiently characterized [3,5,27]. In this study, LC-MS/MS defined the protein-group sequence space used for peptide mining. This proteomics-guided design differs from database-only virtual screening because each theoretical candidate originates from a parental protein group detected in the analyzed material. The main contribution of the workflow is therefore the traceable progression from an experimentally observed proteome to a small set of structurally prioritized peptide candidates [27,28].

Functional annotation was used to characterize the detected protein pool rather than to infer pharmacological action. The presence of collagen-related domains, extracellular-matrix components, cytoskeletal proteins, and proteins involved in processing and metabolism provides context for the sequence resource available for peptide generation. These category-level annotations do not demonstrate that Deer Horn Glue regulates the corresponding pathways, and the downstream RANKL analysis was therefore based on peptide-level prioritization rather than pathway enrichment.

In silico tryptic digestion generated 24,575 nonredundant sequences, which were reduced to 49 candidates by sequential molecular-mass, activity, toxicity, allergenicity, hemolysis, and solubility filters. These prediction tools functioned as triage steps: passing several filters does not establish biological activity or safety, but it substantially reduces the sequence space that must be examined experimentally. The final candidates remain theoretical digestion products until their presence in Deer Horn Glue or a relevant digestion system is confirmed by targeted mass spectrometry. A sequence-matched MS/MS assignment for HEFSVDMTCEGCSNAVTR was observed in the laboratory tryptic digest, although this does not establish its pre-existing occurrence in the product.

RANKL is a key regulator of osteoclast differentiation and bone resorption and represents an important therapeutic target in postmenopausal osteoporosis [29]. HPEPDOCK provided an initial global ranking, and interface-focused Vina analysis of all 49 candidates was added to test the robustness of candidate selection. The modest agreement between the two rankings identified complementary interface-focused candidates while retaining GASLQDWDFGK as the leading sequence. Docking controls further showed that the two scoring stages differed in sequence discrimination. HPEPDOCK, Vina, AlphaFold 3, molecular dynamics, and MM/GBSA thus probe different features of the same computational hypothesis rather than providing independent experimental validation.

Across the five simulated complexes, Lys181, Lys205, Lys244, and Lys257 repeatedly emerged as favorable RANKL contributors, indicating partially overlapping recognition of the receptor surface. This recurrence is mechanistically interesting because the Vina search was centered on a surface containing residues from the RANKL-OPG interface. It should not, however, be equated with inhibition of RANKL-RANK signaling: occupancy of an OPG-related surface could have different functional consequences depending on its overlap with RANK- and OPG-contact sites. The present calculations therefore support a testable interface hypothesis rather than a defined inhibitory mechanism. Direct comparison with established RANK and OPG contact maps, followed by competitive binding experiments, will be needed to determine the functional consequence of peptide occupancy at this surface.

AlphaFold 3 added an orthogonal structural perspective but also exposed uncertainty in the peptide interfaces. GASLQDWDFGK had the strongest relative interface metrics among the five candidates (ipTM = 0.52; mean interchain PAE = 14.07 Å), yet its interface confidence remained moderate. Its agreement with the docking priority therefore supports selection for further testing without establishing the native structure of a GASLQDWDFGK-RANKL complex. For short and flexible peptides, this distinction is particularly important because small changes in pose can alter both contacts and downstream energy estimates.

The independent molecular dynamics simulations and MM/GBSA results illustrate why candidate ranking should not rely on a single descriptor. GASLQDWDFGK showed the most reproducibly low peptide RMSD, whereas HEFSVDMTCEGCSNAVTR formed the largest mean number of interfacial hydrogen bonds. These two candidates also generally produced the most favorable entropy-omitted MM/GBSA estimates, although their order varied among simulations. The estimates therefore support qualitative prioritization rather than a definitive affinity ranking, particularly because conformational entropy was omitted and the peptides differ in length and net charge.

Locally relaxed computational alanine substitutions replaced the original side-chain-deletion approximation and used three independently derived structural medoids as the uncertainty unit. Several originally extreme or broadly distributed effects were not reproducible after relaxation, whereas consistently positive positions provided focused hypotheses for experimental mutagenesis. The revised estimates remain model-dependent because mutant-specific production simulations and conformational entropy were not included.

Several limitations define the scope of these findings. First, the proteomic dataset was generated from one laboratory-prepared Deer Horn Glue batch, and species-level authentication of the starting antler material was not documented; batch and species variability therefore remain unknown. Second, the 707 reported entities are MaxQuant protein groups, and the theoretical peptide candidates require targeted detection in the product or a defined digestion system. Third, the general screening thresholds were not RANKL-specific, and global and interface-focused docking rankings showed only modest agreement. Fourth, the three independent simulations began from the same selected docking pose, while AlphaFold 3 interface confidence was low to moderate and MM/GBSA omitted conformational entropy. Finally, direct binding and functional measurements were not performed. These constraints define the present work as a prioritization framework rather than a definitive characterization of RANKL inhibitors.

The next experimental steps are therefore clear. Targeted LC-MS/MS should first determine whether the prioritized sequences are present in Deer Horn Glue itself or are released under a defined digestion condition. Direct interaction with RANKL should then be tested by an orthogonal biophysical assay such as surface plasmon resonance (SPR), biolayer interferometry (BLI), or microscale thermophoresis (MST), ideally together with competition experiments involving RANK and OPG. Only after direct binding is established should effects on RANKL-RANK signaling, osteoclast differentiation, and bone-resorption phenotypes be used to assess biological relevance.

Overall, this study establishes a proteomics-guided, multiscale route from proteins identified in Deer Horn Glue to a focused set of putative RANKL-binding peptides. Expanded interface docking, matched controls, independent molecular dynamics simulations, and locally relaxed alanine substitutions strengthen the reproducibility of the computational prioritization. GASLQDWDFGK and HEFSVDMTCEGCSNAVTR emerged as complementary priorities based on structural persistence, interfacial hydrogen bonding, and model-dependent energetics. The resulting peptide- and residue-level hypotheses provide a focused basis for targeted detection, direct binding measurements, and functional testing.

## 4. Materials and Methods

### 4.1. Samples, Reagents, and Consumables

Deer antler slices were purchased from Shandong Jianlian Shengjia Traditional Chinese Medicine Co., Ltd. (Jinan, China; batch No. 20210301). Deer Horn Glue was prepared according to the procedure described in the 2020 edition of the Chinese Pharmacopoeia. Briefly, 500 g of deer antler slices were crushed and soaked in a round-bottom flask containing distilled water at a material-to-liquid ratio of 1:5 (*w*/*v*) for 1 h. The material was decocted for 3 h, after which the decoction was collected. Water was replenished, and the residue was decocted for a second 3 h cycle. The two decoctions were combined and concentrated using a rotary evaporator until a viscous concentrate was obtained. After cooling to room temperature, the concentrate was pre-frozen and lyophilized for 48 h. The lyophilized product was ground, passed through a 120-mesh sieve, weighed, and stored at −20 °C until use. A single prepared batch was used for the proteomic and computational analyses; species-level authentication of the starting antler material was not documented. Sodium dodecyl sulfate (SDS) was purchased from Bio-Rad Laboratories (Hercules, CA, USA); tris(hydroxymethyl)aminomethane (Tris), dithiothreitol (DTT; Cat. No. 9163-5G), and iodoacetamide (IAA; Cat. No. 900335-25G) were purchased from Sigma-Aldrich (St. Louis, MO, USA). The bicinchoninic acid (BCA) Protein Assay Kit was purchased from Beyotime Biotechnology (Shanghai, China; Cat. No. P0012), and sequencing-grade trypsin was obtained from Promega (Madison, WI, USA; Cat. No. V5113). Protein electrophoresis was performed using 4–20% precast gradient polyacrylamide gels, with Coomassie Brilliant Blue R-250 for gel staining. Filter-aided sample preparation used ultrafiltration devices for buffer exchange, and digested peptides were desalted using C18 solid-phase extraction material. Acetonitrile and formic acid used for liquid chromatography–mass spectrometry (LC-MS) analysis were LC-MS grade, and experimental water was prepared using a Milli-Q ultrapure water system (Merck Millipore, Burlington, MA, USA). Peptides were loaded using Evotip Standard tips (Evosep Biosystems, Odense, Denmark).

### 4.2. Major Instruments, Software, and Computational Platforms

Peptides were separated using an Evosep One liquid chromatography system (Evosep Biosystems, Odense, Denmark) and introduced through a CaptiveSpray ion source into a timsTOF Pro ion-mobility time-of-flight mass spectrometer (Bruker Daltonics, Bremen, Germany). Raw mass-spectrometry data were processed with MaxQuant 1.6.14.0. Molecular docking was performed using AutoDock Vina 1.2.7, with Open Babel and RDKit used for structural preprocessing and format conversion. Molecular dynamics simulations were conducted using GROMACS 2026.2 with the AMBER ff14SB force field. Data processing and figure generation were performed in R 4.6.1.

### 4.3. Protein Extraction from Deer Horn Glue and Sodium Dodecyl Sulfate–Polyacrylamide Gel Electrophoresis (SDS-PAGE) Analysis

The Deer Horn Glue sample was lysed and proteins were extracted using a buffer containing 4% (*w*/*v*) SDS and 100 mmol/L Tris-HCl at pH 7.6.. Total protein concentration was determined using the BCA assay. A 20 μg aliquot of total protein was mixed with an appropriate volume of 5× protein loading buffer and heated in a boiling-water bath for 5 min. Samples were loaded onto 4–20% precast gradient polyacrylamide gels and electrophoresed at a constant 180 V for 45 min. Gels were stained with Coomassie Brilliant Blue R-250 and imaged. SDS-PAGE was used to assess protein-extraction quality and relative molecular-mass distribution.

### 4.4. Filter-Aided Sample Preparation (FASP) Digestion and Peptide Preparation

Proteins were digested using the filter-aided sample preparation (FASP) [30] method. After reduction with DTT, cysteines were alkylated with IAA, and samples were transferred to ultrafiltration devices. SDS, urea, and other low-molecular-mass components were removed by centrifugal buffer exchange. Sequencing-grade trypsin was then added for digestion. The resulting peptides were desalted using C18 solid-phase extraction material, vacuum freeze-dried, and reconstituted in 40 μL of 0.1% (*v*/*v*) aqueous formic acid. Peptide concentration was estimated from absorbance at 280 nm.

### 4.5. Evosep One Parallel Accumulation–Serial Fragmentation Tandem Mass Spectrometry (PASEF-MS/MS) Analysis

Peptides were loaded onto Evotip Standard tips and separated using the Evosep One system with the 30 samples per day method, using an approximately 44 min gradient. Eluting peptides were introduced into the timsTOF Pro mass spectrometer through a CaptiveSpray ion source. The instrument was operated in positive-ion data-dependent acquisition (DDA) mode with a spray voltage of 1.5 kV and a scan range of *m*/*z* 100–1700. Precursors and fragment ions separated by ion mobility were acquired using parallel accumulation-serial fragmentation (PASEF) [31]. The ion-mobility range was set to 1/K_0_ 0.75–1.35 V·s·cm^−2^ with a cycle time of 0.94 s. Up to 10 PASEF MS/MS scans were acquired per MS1 cycle, with a dynamic exclusion time of 24 s.

### 4.6. Database Searching and Protein Identification

Raw mass-spectrometry data were searched using MaxQuant 1.6.14.0 [32] against a UniProt Knowledgebase (UniProtKB)-derived cervid protein sequence database downloaded on 25 April 2024 [33,34]. The database contained 49,309 target sequence entries, comprising 123 reviewed and 49,186 unreviewed entries. Inspection of the FASTA organism fields showed that this was a broad cervid-related collection rather than a single-species database, with principal entries from *Muntiacus reevesi* and *Cervus* taxa. The exact protein sequence database used for searching is available in the associated data repository. Reverse decoys were generated in revert mode, and the MaxQuant common-contaminant database was included. Protease specificity was set to Trypsin/P with up to two missed cleavages; the minimum peptide length was 7 residues and the maximum peptide mass was 4600 Da. Carbamidomethylation of cysteine was fixed, whereas methionine oxidation and protein N-terminal acetylation were variable modifications. The trapped ion mobility spectrometry–data-dependent acquisition (TIMS-DDA) MS/MS tolerance was 0.05 Da. False discovery rates (FDRs) at the peptide-spectrum-match, peptide, protein-group, and modificationsite levels were controlled at 1%. Minimum peptide, razor-peptide, and unique-peptide counts were 1, 1, and 0, respectively. Match between runs was enabled with a 2 min matching window and a 20 min alignment window; the corresponding ion-mobility windows were 0.05 and 1. Intensity-based absolute quantification (iBAQ) and label-free quantification (LFQ) were enabled, the minimum LFQ ratio count was 1, and LFQ features were required to have MS/MS evidence.

### 4.7. Protein Functional Annotation and Classification

Functional annotation was performed on proteins passing the 1% FDR quality-control threshold. Gene Ontology (GO) annotations were classified into biological process (BP), molecular function (MF), and cellular component (CC) and summarized at GO level 2 (https://geneontology.org/, accessed on 7 May 2024) [35,36]. Identified proteins were further mapped to the Kyoto Encyclopedia of Genes and Genomes (KEGG) database (https://www.kegg.jp/ accessed on 7 May 2024, [37,38,39]), and the number of annotated proteins in each pathway was tabulated. Protein domains were classified using InterPro/Pfam-compatible annotations (https://www.ebi.ac.uk/interpro/, 7 May 2024) [40,41], and subcellular localization was summarized from CELLO predictions (http://cello.life.nctu.edu.tw/, accessed on 7 May 2024) [42].

### 4.8. In Silico Digestion and Candidate-Peptide Library Construction

Database sequences associated with the initial 707 target protein groups were submitted to ExPASy PeptideMass [43] (https://web.expasy.org/peptide_mass/; accessed 11 April 2026) using the Trypsin option with zero missed cleavages. Under this rule, cleavage occurs C-terminal to Lys or Arg except when the following residue is Pro; this differs from MaxQuant Trypsin/P, which permits cleavage before Pro and allowed up to two missed cleavages. Monoisotopic [M+H]^+^ values were selected; cysteine was unmodified in reduced form, and acrylamide adducts and methionine oxidation were not selected. The archived export contained 27,127 peptide records and seven non-sequence modification-annotation rows. After the seven annotation rows were excluded, embedded whitespace was removed and exact duplicate sequences were collapsed, yielding 24,575 nonredundant theoretical peptides.

### 4.9. Candidate-Peptide Activity and Computational Safety Screening

The nonredundant peptide library was first restricted to sequences with predicted molecular masses of 1000–2000 Da. This pragmatic range was selected to retain peptides sufficiently long to provide diverse side-chain contacts for sequence-specific protein–peptide interactions while remaining suitable for computational prioritization and structure-based docking. The retained sequences were then screened sequentially for toxicity, preliminary activity, allergenicity, hemolytic potential, high predicted activity, and water solubility. Toxicity was predicted using ToxinPred [44] (https://webs.iiitd.edu.in/raghava/toxinpred/; accessed 11 April 2026), and sequences classified as “Non-Toxin” by the support vector machine (SVM) were retained. Potential bioactivity was assessed using PeptideRanker [45] (http://distilldeep.ucd.ie/PeptideRanker/; accessed 19 May 2026), which assigns scores ranging from 0 to 1. A permissive cutoff of >0.5 was initially applied to reduce the premature exclusion of potentially bioactive peptides. Allergenicity was predicted using AllerTOP v.2 [46] (https://www.ddg-pharmfac.net/AllerTOP/; accessed 29 June 2026), and sequences predicted as “NON-ALLERGEN” were retained. Hemolytic potential was predicted locally using HemoPI2.0 [47] with the Hybrid2 model, which combines ESM2-t6 and MERCI scores, and the implemented default decision threshold of 0.58. The ESM, MERCI, and hybrid scores were recorded, and sequences classified as “Non-Hemolytic” in the final prediction output were retained. Among the peptides that passed these filters, a more stringent PeptideRanker cutoff of ≥0.9 was then applied to define the high-confidence activity subset. PepCalc [48] (https://pepcalc.com/; accessed 1 July 2026) was used to calculate relative molecular mass, theoretical isoelectric point, net charge at pH 7.0, extinction coefficient, and predicted water solubility; only sequences classified as having “Good water solubility” were retained. This sequential procedure yielded the final set of 49 candidate peptides used in the subsequent docking analyses. The molecular-mass window and PeptideRanker cutoffs were used as pragmatic prioritization criteria rather than empirically optimized thresholds, and no threshold-sensitivity analysis was performed. All screening criteria were applied solely for computational prioritization and do not constitute experimental evidence of biological activity or safety.

### 4.10. HPEPDOCK Global Peptide–Protein Docking

The human RANKL crystal structure was obtained from the Research Collaboratory for Structural Bioinformatics Protein Data Bank (RCSB PDB) [49] (PDB ID: 3URF; https://www.rcsb.org/structure/3URF; accessed 2 July 2026), and chain A was selected as the receptor [50]. The 49 candidate peptides were individually submitted to the HPEPDOCK 2.0 server [51] (http://huanglab.phys.hust.edu.cn/hpepdock/; accessed 5 July 2026) for global peptide–protein docking without a predefined site. Ten models per peptide were retained and ranked by the rank-1 score. The five highest-ranked peptides were predefined for subsequent multiscale analysis, and all 49 candidates were additionally evaluated by the same interface-focused Vina protocol to assess the robustness of this selection.

### 4.11. AutoDock Vina Redocking and Representative-Pose Selection

All 49 candidates and the docking controls were evaluated in a predefined RANKL-interface box using AutoDock Vina 1.2.7 [52]. RANKL residues 162–317 of chain A from 3URF were retained, and the docking box was centered at x = −0.839 Å, y = 2.439 Å, and z = 22.966 Å, with dimensions of 30.55 × 42.00 × 42.00 Å^3^. The search region covered the RANKL-OPG interface residues described previously [50]. For each sequence, three structurally distinct HPEPDOCK conformers were standardized, minimized using the universal force field (UFF) for up to 2000 iterations, and docked with a rigid receptor and rigid peptide conformer (exhaustiveness 32, 20 modes, energy range 10 kcal/mol). Poses were pooled and ranked by Vina score; the best-scoring pose was used to initiate molecular dynamics. The matched control set comprised 15 composition-preserving scrambled peptides, 10 interface-derived sequences, and the literature peptide YR-11. YR-11 (YLEIEFSLKHR), an experimentally reported OPG-derived RANKL-targeting peptide [53], was included as a literature reference. Group differences were assessed using one-sided Mann–Whitney U tests, with more-negative scores specified as the alternative and tie-aware exact label permutations used for small comparisons. To account for matching, each candidate was additionally compared with the mean of its three composition-preserving scrambled controls using a one-sided exact Wilcoxon signed-rank test (*n* = 5 pairs). This protocol samples several starting conformers but does not represent full peptide flexibility or receptor induced fit; Vina scores were used for pose selection rather than as experimental binding energies.

### 4.12. AlphaFold 3 Complex-Structure Prediction

AlphaFold Server (https://alphafoldserver.com/; accessed 23 August 2026) was used to generate five AlphaFold 3 models for each of the five initially prioritized peptide–RANKL systems [54]. Each system contained the 156-residue RANKL chain and the candidate peptide; no small-molecule ligands, metal ions, or covalent modifications were included, and other settings were left at default. ipTM, pTM, ranking score, and the PAE matrix were recorded for the top-ranked model. Mean and median bidirectional interchain PAE values were calculated across all residue pairs spanning the peptide and RANKL chains and were emphasized over the minimum pairwise value.

### 4.13. Molecular Dynamics Simulations

For each of the five peptide–RANKL systems, the same selected Vina pose was used to initiate three independently seeded 200 ns all-atom MD simulations in GROMACS 2026.2 [55] giving 15 production trajectories. AMBER ff14SB [56] and TIP3P water were used in a dodecahedral box with a minimum solute distance of 1.0 nm and 0.15 mol/L NaCl. After steepest-descent minimization, 100 ps constant-number, constant-volume, and constant-temperature (NVT) equilibration and 100 ps constant-number, constant-pressure, and constant-temperature (NPT) equilibration were performed [57]. Production used a 2 fs step, linear constraint solver (LINCS) constraints, particle mesh Ewald (PME) electrostatics, V-rescale temperature coupling at 298 K, and isotropic C-rescale pressure coupling at 1 bar with tau-p = 2.0 ps. Independent initial-velocity seeds are listed in Table S6. Coordinates and energies were saved every 20 ps [58].

### 4.14. Molecular Dynamics Trajectory Analysis

Each trajectory was processed independently after periodic-boundary correction and fitting to the RANKL backbone. Peptide root-mean-square deviation (RMSD), RANKL root-mean-square fluctuation (RMSF), complex radius of gyration (Rg), complex solvent-accessible surface area (SASA), peptide–RANKL hydrogen bonds, and minimum distance were calculated. Complete-trajectory and 150–200 ns means were first calculated for each trajectory; the three trajectory means were then summarized as mean ± sample SD. Time frames were not treated as independent replicates.

### 4.15. MM/GBSA Binding Free-Energy Calculation and Residue Energy Decomposition

For each independent 200 ns production trajectory, 101 snapshots were sampled at 2 ns intervals for a single-trajectory OBC-II/SASA MM/GBSA endpoint calculation. Each independent trajectory supplied one mean estimate; across-simulation uncertainty was reported as the sample SD of the three trajectory means [59].

Binding free energy was calculated as follows:

$$\Delta G_{bind}=\Delta E_{vdW}+\Delta E_{elec}+\Delta G_{GB}+\Delta G_{SA}$$

where $\Delta E_{vdW}$ represents the van der Waals interaction-energy contribution, $\Delta E_{elec}$ the electrostatic interaction-energy contribution, $\Delta G_{GB}$ the polar solvation free energy, and $\Delta G_{SA}$ the nonpolar solvation free energy.

The polar term used OBC-II (igb = 5), mbondi2 radii, and solute/solvent dielectric constants of 1/80. The nonpolar term used Shrake–Rupley SASA with a 0.14 nm probe, 960 Fibonacci-sphere points per atom, and gamma = 2.267 kJ mol^−1^ nm^−2^. Conformational entropy was not calculated. Values are therefore described as entropy-omitted endpoint estimates and used only for qualitative comparison. Residue decomposition was analyzed separately for each independent simulation [60].

### 4.16. Computational Alanine Scanning

One peptide-backbone structural medoid was selected from 150–200 ns of each independent replicate simulation. Each scannable residue was replaced with a standard AMBER ff14SB alanine residue. Wild-type (WT) and mutant receptor-peptide systems were minimized independently with OpenMM using the Onufriev–Bashford–Case II (OBC-II) implicit-solvent model, a tolerance of 10 kJ mol^−1^ nm^−1^ and a maximum of 1000 iterations [61,62].

The change in binding free energy induced by each residue mutation was calculated as follows:

$$\Delta \Delta G_{i}=\Delta G_{bind}^{i\to Ala}-\Delta G_{bind}^{WT}$$

where $\Delta G_{bind}^{i\to Ala}$ is the predicted binding free energy after mutation of residue *i* to alanine, $G_{bind}^{WT}$ is the predicted binding free energy of the corresponding wild-type complex. When $\Delta \Delta G_{i}$ > 0, alanine substitution is predicted to reduce binding stability, indicating that the original side chain may favor complex stabilization; when $\Delta \Delta G_{i}$ < 0, the mutation is predicted to favor binding under the current computational model.

Means and sample SDs were calculated from the three independently derived medoid estimates. No mutant-specific production MD or conformational-entropy calculation was performed; values are locally relaxed, model-dependent sensitivity estimates rather than experimental mutation free energies [63].

### 4.17. Data Processing and Statistical Analysis

Online prediction scores and docking scores were used descriptively. For MD observables, MM/GBSA, and locally relaxed alanine substitutions, the independent 200 ns simulation was the replicate unit and results were summarized as mean ± sample SD across three simulation-level values. Framewise standard deviation (SD) or standard error of the mean (SEM) was used only to describe correlated temporal fluctuations within a trajectory. No hypothesis-testing *p* values were calculated for the three-simulation structural summaries.

## 5. Conclusions

In this study, proteomic analysis combined with multiscale computational approaches prioritized five putative RANKL-binding peptides from proteins identified in Deer Horn Glue. Expanded interface docking and matched controls tested the robustness of the initial selection, while three independent molecular dynamics simulations, entropy-omitted MM/GBSA, and locally relaxed alanine substitutions provided a more conservative assessment of structural and energetic consistency. GASLQDWDFGK and HEFSVDMTCEGCSNAVTR emerged as complementary priorities for targeted detection, direct RANKL-binding measurements, and functional validation.

## Figures and Tables

**Table 2:** AlphaFold 3 confidence metrics of the predicted candidate peptide–RANKL complexes. Abbreviations: RANKL, receptor activator of nuclear factor-κB ligand; ipTM, interface predicted template modeling score; pTM, predicted template modeling score; PAE, predicted aligned error.

Peptide	ipTM	pTM	Ranking Score	Minimum Interchain PAE (Å)	Mean Interchain PAE (Å)	Median Interchain PAE (Å)
GASLQDWDFGK	0.52	0.89	0.63	4.09	14.07	12.25
GGYEDPYYGYDDGYAVR	0.33	0.86	0.48	9.00	19.20	18.20
DGAADWWLGVAGDMFR	0.42	0.86	0.55	5.00	16.60	15.30
HEFSVDMTCEGCSNAVTR	0.20	0.83	0.38	12.45	22.10	22.40
NWWTDSSAEK	0.44	0.89	0.56	4.79	15.60	13.90

**Table 4:** Entropy-omitted MM/GBSA endpoint binding-energy estimates for the five candidate peptide–RANKL complexes from three independently seeded simulations. Abbreviations: MM/GBSA, molecular mechanics/generalized Born surface area; ΔGbind, binding free energy; SD, standard deviation.

Peptide	Simulation 1 Mean ΔGbind (kcal/mol)	Simulation 2 Mean ΔGbind (kcal/mol)	Simulation 3 Mean ΔGbind (kcal/mol)	Across-Simulation Mean ± Sample SD (kcal/mol)
GASLQDWDFGK	−144.22	−181.84	−207.04	−177.70 ± 31.61
GGYEDPYYGYDDGYAVR	−150.93	−71.76	−38.01	−86.90 ± 57.96
DGAADWWLGVAGDMFR	−79.28	−50.37	0.46	−43.06 ± 40.37
HEFSVDMTCEGCSNAVTR	−257.26	−209.50	−148.66	−205.14 ± 54.43
NWWTDSSAEK	−63.21	−2.31	−86.13	−50.55 ± 43.32

**Table 5:** Key RANKL residues contributing to peptide binding identified by MM/GBSA decomposition. Abbreviations: RANKL, receptor activator of nuclear factor-κB ligand; MM/GBSA, molecular mechanics/generalized Born surface area.

Rank	GASLQDWDFGK	GGYEDPYYGYDDGYAVR	DGAADWWLGVAGDMFR	HEFSVDMTCEGCSNAVTR	NWWTDSSAEK
1	Lys282 (−50.75)	Lys181 (−53.27)	Lys244 (−56.68)	Lys257 (−63.74)	Lys181 (−15.18)
2	Lys244 (−50.03)	Ser251 (−27.74)	Lys282 (−56.25)	Lys181 (−58.06)	Pro176 (−14.50)
3	Lys248 (−42.90)	Lys257 (−23.37)	Lys257 (−53.86)	Lys205 (−22.78)	Lys205 (−14.25)
4	Arg284 (−33.06)	Lys244 (−19.40)	Arg284 (−44.25)	Ser251 (−13.23)	Ile175 (−13.81)
5	Lys257 (−26.81)	Lys205 (−18.61)	Lys262 (−37.87)	Lys244 (−13.20)	Ser177 (−12.57)

## Data Availability

The mass spectrometry proteomics data, MaxQuant output files, and the exact FASTA database used for database searching are available via ProteomeXchange through the PRIDE repository under accession PXD083344. Peptide-screening tables, docking inputs and output structures, scripts and parameter files, molecular-dynamics input systems, analysis-ready trajectories and representative coordinate files, and the underlying MM/GBSA and computational alanine-substitution data are publicly available in Zenodo at 10.5281/zenodo.22680210.

## Supplementary Materials

The following supporting information can be downloaded at: https://www.mdpi.com/article/10.3390/ijms27188255/s1.

## Abbreviations

The following abbreviations are used in this manuscript:

LC-MS/MS	Liquid chromatography-tandem mass spectrometry
DDA	Data-dependent acquisition
PASEF	Parallel accumulation-serial fragmentation
GO	Gene Ontology
KEGG	Kyoto Encyclopedia of Genes and Genomes
FASP	Filter-aided sample preparation
SDS	Sodium dodecyl sulfate
DTT	Dithiothreitol
IAA	Iodoacetamide
RANKL	Receptor activator of nuclear factor-κB ligand
RANK	Receptor activator of nuclear factor-κB
OPG	Osteoprotegerin
OP	Osteoporosis
PMOP	Postmenopausal osteoporosis
HPEPDOCK	Hierarchical computational protein–peptide docking
Vina	AutoDock Vina
MD	Molecular dynamics
RMSD	Root mean square deviation
RMSF	Root mean square fluctuation
Rg	Radius of gyration
SASA	Solvent-accessible surface area
MM/GBSA	Molecular mechanics/generalized Born surface area
ΔG-bind	Binding free energy
PAE	Predicted aligned error
ipTM	Interface predicted template modeling score
pTM	Predicted template modeling score
ΔΔG-bind	Difference in binding free energy
SEM	Standard error of the mean
MW	Molecular weight
pI	Isoelectric point
LFQ	Label-free quantification
iBAQ	Intensity-based absolute quantification
FDR	False discovery rate
PSM	Peptide-spectrum match
PDB	Protein Data Bank
