# Supplementary material for: Proteomics-Guided Computational Prioritization of Putative RANKL-Binding Peptides from Proteins Identified in Deer Horn Glue
Source: Int J Mol Sci. 2026 Sep 16;27(18):8255. doi: 10.3390/ijms27188255 (PMC13607427; doi:10.3390/ijms27188255)
Supplement: Supplementary file 1 [file ijms-27-08255-s001.zip › ijms-4560440-supplementary.pdf]

Supplementary Materials

Proteomics-Guided Computational Prioritization of Putative RANKL-Binding Peptides from Proteins Identified in Deer Horn Glue

Zhonghao Fan, Tiefeng Sun, Heng Aik Teng, Haitao Du, Kun Yang, Cheng Wang, Jingwen Ma and Ping Wang

Contents

- Figure S1. Extended quality distributions for protein and peptide identifications.
- Figure S2. Representative annotated MS/MS spectra from the Deer Horn Glue proteome.
- Figure S3. Representative R1 minimum peptide-RANKL distance trajectories.
- Figure S4. R2 molecular-dynamics and entropy-omitted MM/GBSA profiles.
- Figure S5. R3 molecular-dynamics and entropy-omitted MM/GBSA profiles.
- Figure S6. MM/GBSA estimates across three independent trajectories.
- Figure S7. R2 residue-wise MM/GBSA energy decomposition.
- Figure S8. R3 residue-wise MM/GBSA energy decomposition.
- Table S1. Screening properties and global and interface-focused docking rankings for all 49 retained candidates.
- Table S2. Starting conformers and representative AutoDock Vina poses.
- Table S3. Extended AlphaFold 3 interface-confidence metrics.
- Table S4. Entropy-omitted MM/GBSA estimates over consecutive 15-ns windows for R1-R3.
- Table S5. Locally relaxed alanine-substitution estimates across R1-R3 trajectory medoids.
- Table S6. Molecular-dynamics protocol and random seeds.
- Table S7. Replicate-level summary of molecular-dynamics observables.
- Table S8. Full MM/GBSA summary across the three independent trajectories.

Associated machine-readable files are publicly available in the Zenodo repository cited in the Data Availability Statement: Data S1, complete list of the 707 retained target protein groups; Data S2, complete list of the 2968 experimentally identified peptide sequences; and Data S3, machine-readable R1-R3 trajectory-level MD, MM/GBSA, and locally relaxed alanine-substitution summaries.

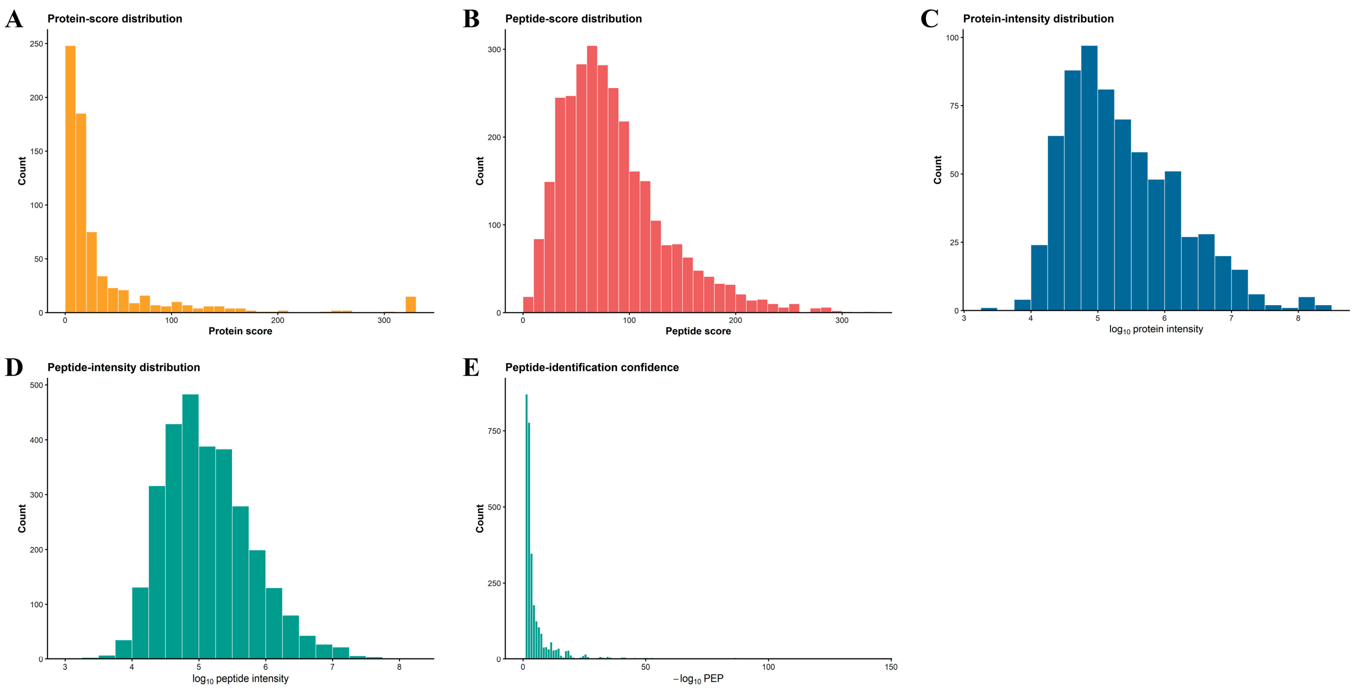

Figure S1. Extended quality distributions for protein and peptide identifications in the Deer Horn Glue proteome. (A) Protein-score distribution; (B) peptide-score distribution; (C) protein-intensity distribution; (D) peptide-intensity distribution; and (E) peptide-identification confidence expressed as -log10 posterior error probability (PEP).

A

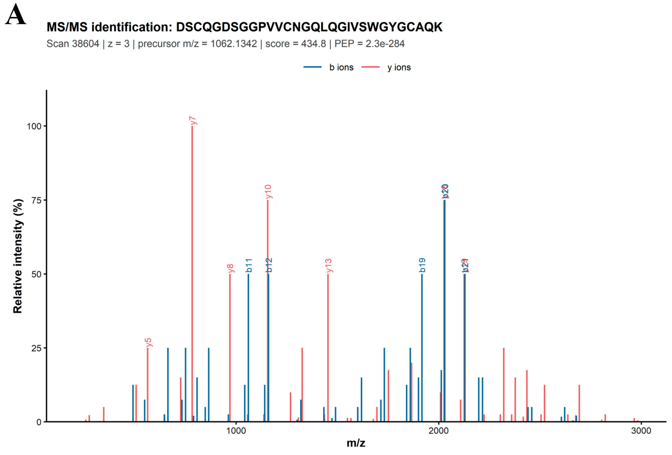

B

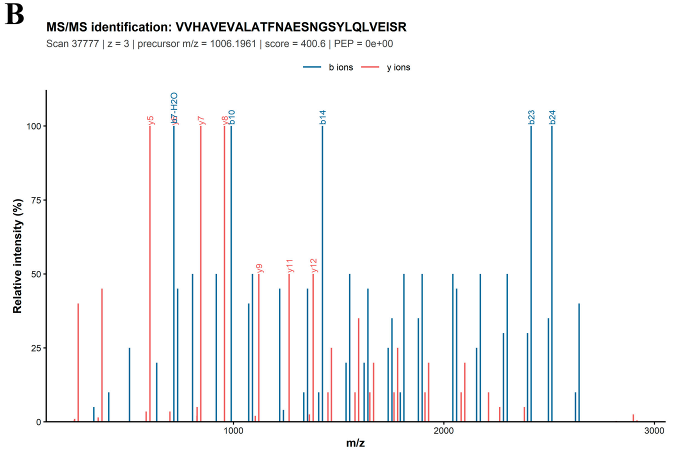

Figure S2 (continued)

C

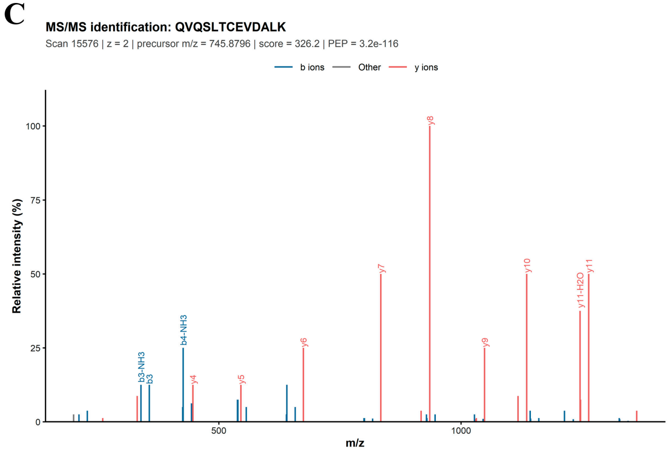

D

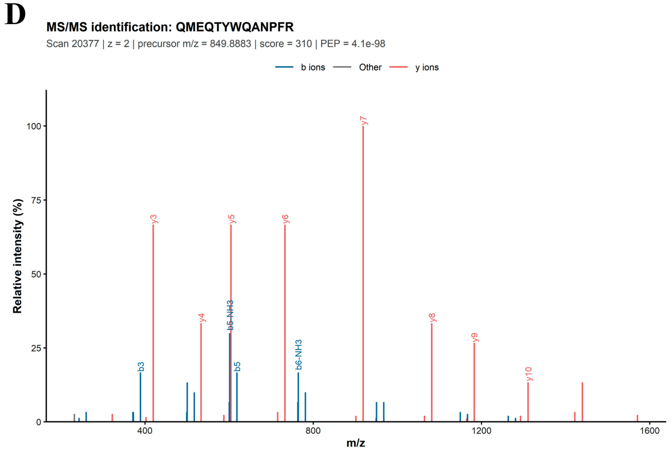

Figure S2 (continued)

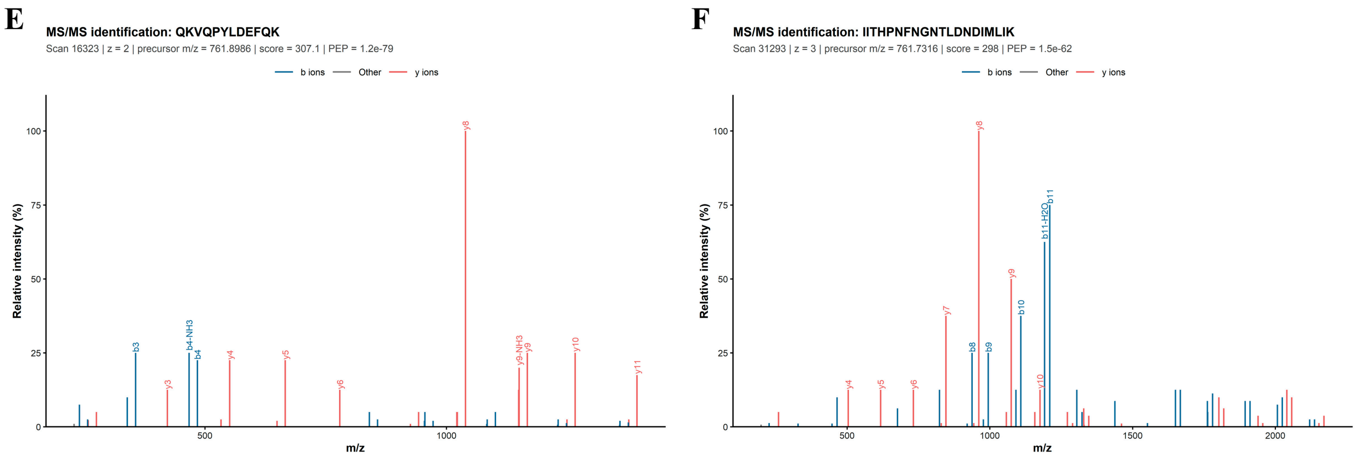

Figure S2. Representative annotated MS/MS spectra from the Deer Horn Glue proteome. (A) DSCQGDSGGPVVCNGQLQGIVSWGYGCAQK; (B) VVHAVEVALATFNAESNGSYLQLVEISR; (C) QVQSLTCEVDALK; (D) QMEQTYWQANPFR; (E) QKVQPYLDEFQK; and (F) IITHPNFNGNTLDNDIMLIK. Blue and red annotations denote matched b- and y-type ions, respectively. These spectra document representative proteomic identifications and should not be interpreted as direct detection of the five in silico-prioritized RANKL-binding candidates.

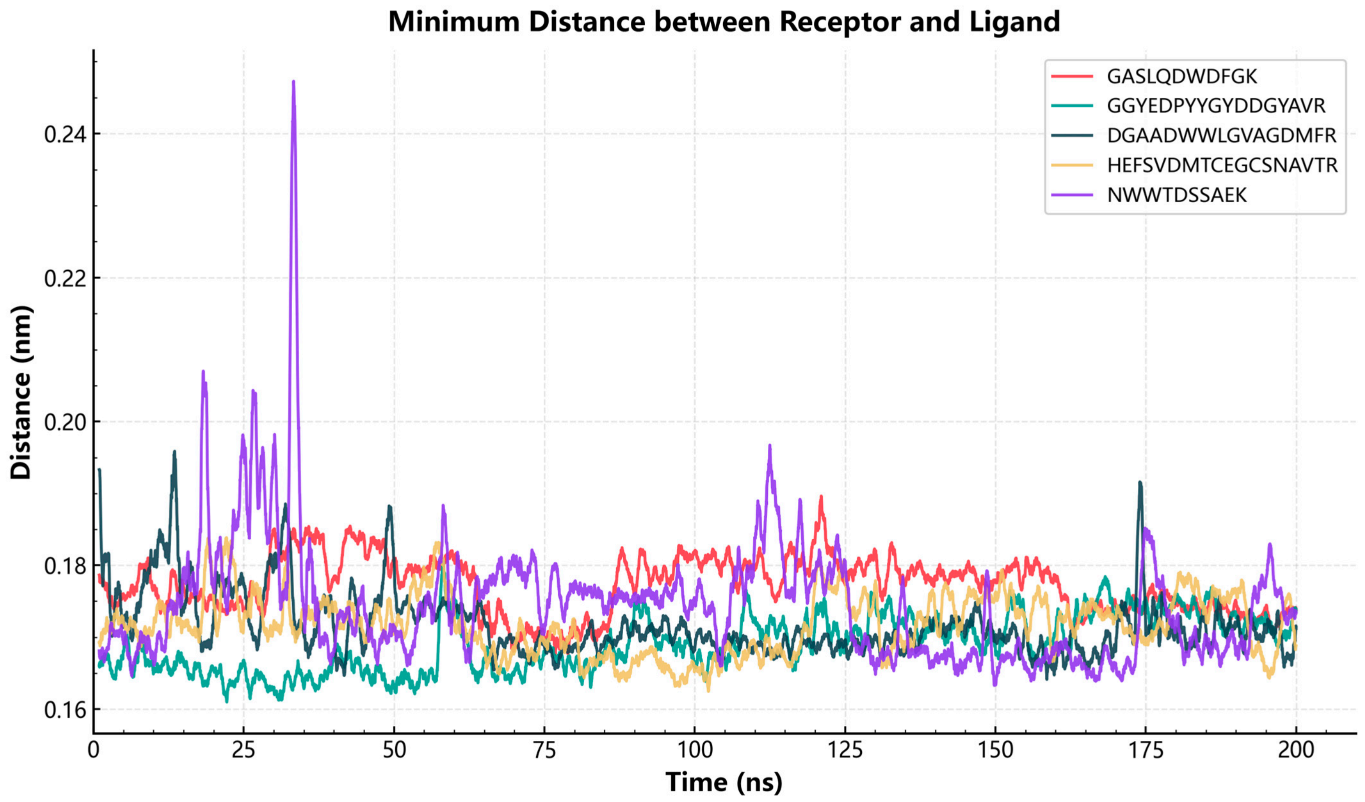

Figure S3. Representative minimum peptide-RANKL distance trajectories from the R1 200-ns molecular-dynamics simulations. The corresponding independently seeded R2 and R3 analyses are summarized in Figures S4-S6 and Tables S4, S7, and S8. Distances were calculated after correction of periodic-boundary artifacts and trajectory fitting.

Table S1. Screening properties and global and interface-focused docking rankings for all 49 retained candidate peptides. *Part A summarizes screening and physicochemical properties; Part B lists the complete rank 1-10 HPEPDOCK scores; and Part C compares the global HPEPDOCK and interface-focused AutoDock Vina scores and rankings. More negative values indicate more favorable scores within the corresponding docking function.*

Rank	Sequence	Rank-1 score	PeptideRanker	Allergenicity	Hemolysis	Solubility	Length	MW (Da)	pI	Charge (pH 7)
1	GASLQDWDFGK	-225.61	0.976	Non-allergenic	Non-hemolytic	Good	11	1223.29	3.71	-1
2	GGYEDPYYGYDDGYAVR	-224.94	0.95	Non-allergenic	Non-hemolytic	Good	17	1959.97	3.32	-3
3	DGAADWWLGVAGDMFR	-219.08	0.946	Non-allergenic	Non-hemolytic	Good	16	1766.93	3.41	-2
4	HEFSVDMTCEGCSNAVTR	-214.70	0.987	Non-allergenic	Non-hemolytic	Good	18	1986.18	4.31	-2
5	NWWTDSSAEK	-208.41	0.977	Non-allergenic	Non-hemolytic	Good	10	1223.25	3.93	-1
6	YDGNCAEQDGSGWWMNK	-205.18	0.992	Non-allergenic	Non-hemolytic	Good	17	1961.06	3.54	-2.1
7	GHYTEGAELVDSVLDVVR	-202.75	0.999	Non-allergenic	Non-hemolytic	Good	18	1959.12	3.93	-2.9
8	VAPAPARPSGPSK	-202.44	0.989	Non-allergenic	Non-hemolytic	Good	13	1234.41	11.4	2
9	QVIPGLEQSLLDMCVGEK	-200.15	0.988	Non-allergenic	Non-hemolytic	Good	18	1959.29	3.69	-2.1
10	GEIGFPGRPGRPGMNGLK	-199.63	0.918	Non-allergenic	Non-hemolytic	Good	18	1840.12	11.23	2
11	DGVGQEPVHLESPAQEHR	-196.90	0.902	Non-allergenic	Non-hemolytic	Good	18	1985.08	4.51	-2.8
12	VTWEPAPGEVK	-196.02	0.926	Non-allergenic	Non-hemolytic	Good	11	1212.35	4.15	-1
13	FYFVGDEDLLEIIGNSK	-195.48	0.979	Non-allergenic	Non-hemolytic	Good	17	1959.15	3.43	-3
14	SSTPVPPPPSLR	-194.87	0.985	Non-allergenic	Non-hemolytic	Good	12	1234.40	10.57	1
15	NMADQIIQEIYGQIQSK	-192.96	0.979	Non-allergenic	Non-hemolytic	Good	17	1979.22	3.93	-1
16	DLETQVIQLNEQVHSLK	-192.76	0.917	Non-allergenic	Non-hemolytic	Good	17	1994.21	4.31	-1.9
17	HRPELIDYGK	-192.50	0.975	Non-allergenic	Non-hemolytic	Good	10	1227.37	7.54	0.1
18	TATVYPEPPNK	-192.06	0.912	Non-allergenic	Non-hemolytic	Good	11	1216.34	6.54	0
19	MQLGGRPPVPR	-191.94	0.941	Non-allergenic	Non-hemolytic	Good	11	1207.45	12.1	2
20	LSPFMADIR	-189.04	0.995	Non-allergenic	Non-hemolytic	Good	9	1049.25	6.64	0
21	GRPVGFPMR	-188.36	0.912	Non-allergenic	Non-hemolytic	Good	9	1016.22	12.1	2
22	SPPSPCDAAPFR	-187.24	0.931	Non-allergenic	Non-hemolytic	Good	12	1244.38	5.9	-0.1
23	LHDVDFYK	-186.28	0.92	Non-allergenic	Non-hemolytic	Good	8	1036.14	5.04	-0.9
24	EAFNMIDQNR	-183.52	0.94	Non-allergenic	Non-hemolytic	Good	10	1237.34	3.93	-1
25	VCINSEHSVDTLLETLGK	-182.11	0.991	Non-allergenic	Non-hemolytic	Good	18	1958.20	4.31	-2
26	QNPSNPLQR	-181.86	0.953	Non-allergenic	Non-hemolytic	Good	9	1053.13	10.55	1
27	DGPPGLPGPPGPPGTPGK	-181.69	0.927	Non-allergenic	Non-hemolytic	Good	18	1594.77	6.66	0
28	GECPRPNHTAK	-181.59	0.978	Non-allergenic	Non-hemolytic	Good	11	1209.34	8.84	1

Rank	Sequence	Rank-1 score	PeptideRanker	Allergenicity	Hemolysis	Solubility	Length	MW (Da)	pI	Charge (pH 7)
29	HIDLVEGDEGR	-180.95	0.984	Non-allergenic	Non-hemolytic	Good	11	1239.29	3.93	-2.9
30	CLDGYIGDAIR	-177.45	0.938	Non-allergenic	Non-hemolytic	Good	11	1195.35	3.71	-1.1
31	IQLINNMLDK	-175.53	0.982	Non-allergenic	Non-hemolytic	Good	10	1201.44	6.64	0
32	DVTFSPASIEK	-173.83	0.914	Non-allergenic	Non-hemolytic	Good	11	1193.30	3.93	-1
33	VVMDGVISDEECQELQR	-173.43	0.959	Non-allergenic	Non-hemolytic	Good	17	1950.16	3.34	-4.1
34	MLEAYNLTEK	-171.04	0.97	Non-allergenic	Non-hemolytic	Good	10	1211.39	4.15	-1
35	ATGPPVSELITK	-168.76	0.986	Non-allergenic	Non-hemolytic	Good	12	1212.39	6.91	0
36	AETLNPTPVTAK	-167.28	0.906	Non-allergenic	Non-hemolytic	Good	12	1241.39	6.91	0
37	ILESINESALK	-161.91	0.97	Non-allergenic	Non-hemolytic	Good	11	1216.38	4.15	-1
38	EVFEDAVEIR	-160.84	0.91	Non-allergenic	Non-hemolytic	Good	10	1206.30	3.54	-3
39	GGSGSGPTIEEVD	-157.65	0.908	Non-allergenic	Non-hemolytic	Good	13	1204.20	0.76	-3
40	VDEETENTMR	-145.68	0.948	Non-allergenic	Non-hemolytic	Good	10	1223.27	3.54	-3
41	ETIPDIDPNAK	-143.44	0.94	Non-allergenic	Non-hemolytic	Good	11	1212.31	3.54	-2
42	LEDELVAQK	-142.43	0.941	Non-allergenic	Non-hemolytic	Good	9	1044.16	3.69	-2
43	TEALLGDEQAR	-141.21	0.911	Non-allergenic	Non-hemolytic	Good	11	1202.27	3.69	-2
44	VMDSDEDDDY	-138.86	0.991	Non-allergenic	Non-hemolytic	Good	10	1203.15	0.37	-6
45	EAESECEQLAK	-138.37	0.96	Non-allergenic	Non-hemolytic	Good	11	1236.31	3.67	-3.1
46	SIQEIQELDK	-135.65	0.931	Non-allergenic	Non-hemolytic	Good	10	1202.31	3.69	-2
47	EVEEDEYK	-134.77	0.92	Non-allergenic	Non-hemolytic	Good	8	1040.03	3.44	-4
48	CTDLQEEDLK	-134.03	0.907	Non-allergenic	Non-hemolytic	Good	10	1193.28	3.43	-3.1
49	GSPLEEEGGSLK	-133.81	0.993	Non-allergenic	Non-hemolytic	Good	12	1202.27	3.85	-2

Table S1, Part B. Complete HPEPDOCK rank 1-10 scores.

Overall rank	Sequence	Docking rank 1	Docking rank 2	Docking rank 3	Docking rank 4	Docking rank 5	Docking rank 6	Docking rank 7	Docking rank 8	Docking rank 9	Docking rank 10
1	GASLQDWDFGK	-225.61	-215.91	-203.52	-199.99	-193.81	-192.97	-188.76	-184.34	-182.45	-181.33
2	GGYEDPYYGYDDGYAVR	-224.94	-213.82	-213.47	-209.34	-203.06	-201.21	-200.43	-199.01	-198.84	-198.45
3	DGAADWWLGVAGDMFR	-219.08	-206.96	-204.99	-203.91	-202.39	-199.69	-199.27	-195.93	-194.29	-191.49
4	HEFSVDMTCEGCSNAVTR	-214.70	-211.97	-193.44	-189.31	-184.51	-183.47	-182.18	-180.21	-178.83	-178.22
5	NWWTDSSAEK	-208.41	-188.77	-188.09	-185.71	-185.42	-183.88	-183.78	-182.35	-181.70	-180.64
6	YDGNCAEQDGSGWWMNK	-205.18	-200.52	-200.36	-200.30	-200.19	-199.06	-198.36	-197.10	-196.48	-196.44
7	GHYTEGAELVDSVLDVVR	-202.75	-193.05	-189.12	-186.04	-185.78	-184.78	-184.26	-182.56	-181.23	-180.85
8	VAPAPARPSGPSK	-202.44	-192.79	-189.11	-184.14	-180.71	-179.96	-178.54	-178.41	-177.24	-175.22

Overall rank	Sequence	Docking rank 1	Docking rank 2	Docking rank 3	Docking rank 4	Docking rank 5	Docking rank 6	Docking rank 7	Docking rank 8	Docking rank 9	Docking rank 10
9	QVIPGLEQSLLDMCVGEK	-200.15	-198.48	-181.69	-178.26	-173.05	-168.40	-167.90	-167.40	-167.27	-165.41
10	GEIGFPGRPGRPGMNGLK	-199.63	-193.72	-192.24	-186.26	-183.23	-182.14	-181.47	-180.94	-179.55	-178.37
11	DGVGQEPVHLESPAQEHR	-196.90	-182.77	-175.55	-174.24	-173.90	-173.71	-171.57	-171.08	-170.64	-169.55
12	VTWEPAPGEVK	-196.02	-177.92	-177.06	-173.69	-172.39	-172.31	-171.71	-169.79	-169.13	-168.95
13	FYFVGDEDLLEIIGNSK	-195.48	-192.37	-189.46	-181.64	-181.45	-180.62	-177.66	-177.13	-176.65	-176.59
14	SSTPVPPPPSLR	-194.87	-177.70	-176.75	-171.60	-170.48	-170.24	-170.09	-169.35	-168.80	-168.45
15	NMADQIIQEIYGQIQSK	-192.96	-185.92	-179.84	-177.11	-176.69	-175.11	-174.78	-172.55	-172.10	-171.78
16	DLETQVIQLNEQVHSLK	-192.76	-183.23	-169.28	-167.27	-164.42	-163.58	-162.78	-161.15	-161.15	-160.84
17	HRPELIDYGK	-192.50	-179.66	-176.83	-174.96	-173.14	-171.16	-167.42	-166.82	-166.77	-165.87
18	TATVYPEPPNK	-192.06	-187.84	-182.16	-176.37	-174.28	-174.13	-173.67	-173.42	-171.88	-171.71
19	MQLGGRPPVPR	-191.94	-187.79	-184.81	-176.36	-175.78	-175.54	-174.96	-173.01	-172.09	-171.62
20	LSPFMADIR	-189.04	-183.51	-182.37	-174.03	-173.83	-173.35	-171.45	-168.98	-167.54	-167.40
21	GRPVGFPMR	-188.36	-184.72	-182.53	-182.41	-181.95	-180.82	-180.09	-179.29	-178.75	-178.52
22	SPPSPCDAAPFR	-187.24	-184.34	-177.74	-176.36	-175.90	-175.01	-174.21	-173.67	-172.98	-172.66
23	LHDVDFYK	-186.28	-185.90	-180.19	-178.67	-178.32	-178.12	-177.95	-177.35	-175.02	-174.97
24	EAFNMIDQNR	-183.52	-177.56	-174.76	-174.39	-169.59	-166.63	-166.46	-165.60	-165.19	-164.01
25	VCINSEHSVDTLLETLGK	-182.11	-181.72	-178.78	-178.21	-178.03	-175.33	-175.30	-175.19	-174.96	-173.94
26	QNPSNPLQR	-181.86	-168.14	-167.86	-167.81	-165.83	-161.04	-160.78	-158.55	-157.39	-157.00
27	DGPPGLPGPPGPPGTPGK	-181.69	-174.27	-172.51	-168.23	-167.80	-166.92	-163.55	-163.50	-162.46	-162.45
28	GECPRPNHTAK	-181.59	-171.63	-170.01	-169.57	-167.98	-167.67	-166.69	-166.63	-165.12	-163.63
29	HIDLVEGDEGR	-180.95	-155.77	-155.52	-155.09	-152.72	-149.38	-147.47	-142.91	-142.69	-142.07
30	CLDGYIGDAIR	-177.45	-176.31	-175.06	-174.68	-171.73	-165.88	-165.62	-160.66	-160.20	-158.31
31	IQLINNMLDK	-175.53	-163.30	-160.03	-156.85	-152.90	-149.28	-148.79	-146.89	-146.52	-146.33
32	DVTFSPASIEK	-173.83	-168.62	-166.12	-166.03	-164.13	-163.57	-162.58	-162.39	-161.78	-161.59
33	VVMDGVISDEECQELQR	-173.43	-171.86	-162.20	-154.03	-153.47	-152.79	-147.88	-145.67	-145.19	-144.88
34	MLEAYNLTEK	-171.04	-170.71	-167.29	-166.63	-166.52	-164.44	-164.33	-163.44	-162.99	-162.58
35	ATGPPVSELITK	-168.76	-161.60	-159.39	-153.57	-152.66	-150.49	-150.25	-149.96	-149.39	-149.38
36	AETLNPTPVTAK	-167.28	-167.16	-162.13	-161.93	-160.96	-159.85	-159.35	-158.91	-156.70	-156.35
37	ILESINESALK	-161.91	-151.11	-150.91	-149.91	-149.65	-145.83	-145.32	-142.18	-141.80	-140.84
38	EVFEDAVEIR	-160.84	-160.74	-149.60	-148.85	-147.91	-147.57	-146.34	-145.35	-144.33	-144.04
39	GGSGSGPTIEEVD	-157.65	-146.63	-145.11	-142.73	-142.34	-140.95	-140.06	-139.66	-139.27	-138.05

Overall rank	Sequence	Docking rank 1	Docking rank 2	Docking rank 3	Docking rank 4	Docking rank 5	Docking rank 6	Docking rank 7	Docking rank 8	Docking rank 9	Docking rank 10
40	VDEETENTMR	-145.68	-140.35	-137.79	-137.37	-136.01	-135.79	-133.82	-133.80	-130.78	-130.40
41	ETIPDIDPNAK	-143.44	-141.97	-141.12	-141.08	-140.93	-140.83	-140.64	-140.52	-139.29	-138.09
42	LEDELVAQK	-142.43	-137.79	-136.93	-136.76	-136.24	-132.34	-131.67	-130.47	-128.98	-126.57
43	TEALLGDEQAR	-141.21	-141.01	-137.03	-135.34	-135.18	-135.01	-133.10	-131.19	-129.64	-128.78
44	VMDSDEDDDY	-138.86	-133.67	-132.69	-128.72	-127.19	-124.04	-123.87	-122.90	-120.01	-119.22
45	EAESECEQLAK	-138.37	-133.64	-128.95	-127.23	-126.64	-121.22	-120.89	-120.18	-119.09	-118.61
46	SIQEIQELDK	-135.65	-133.52	-131.83	-131.72	-131.15	-130.89	-130.80	-130.71	-130.37	-129.68
47	EVEEDEYK	-134.77	-129.75	-123.67	-123.14	-122.58	-120.79	-119.92	-115.47	-115.38	-115.27
48	CTDLQEEDLK	-134.03	-131.05	-129.84	-125.69	-125.66	-124.29	-123.87	-123.65	-121.81	-120.81
49	GSPLEEEGGSLK	-133.81	-133.43	-132.86	-132.50	-130.06	-129.08	-127.69	-127.64	-127.14	-126.82

Table S1, Part C. Comparison of global HPEPDOCK and interface-focused AutoDock Vina scores and rankings for all 49 retained candidate peptides.

Peptide sequence	HPEPDOCK rank-1 score	HPEPDOCK rank	Interface-focused Vina score (kcal/mol)	Vina rank
GASLQDWDFGK	−225.607	1	−12.076	1
VCINSEHSVDTLLETLGK	−182.110	25	−11.895	2
YDGNCAEQDGSGWWMNK	−205.178	6	−11.533	3
GGYEDPYYGYDDGYAVR	−224.937	2	−11.291	4
DGVGQEPVHLESPAQEHR	−196.897	11	−11.237	5
TATVYPEPPNK	−192.062	18	−11.209	6
GEIGFPGRPGRPGMNGLK	−199.634	10	−11.203	7
DLETQVIQLNEQVHSLK	−192.763	16	−11.136	8
NMADQIIQEIYGQIQSK	−192.956	15	−11.115	9
VVMDGVISDEECQELQR	−173.429	33	−11.058	10
FYFVGDEDLLEIIGNSK	−195.476	13	−10.967	11
CLDGYIGDAIR	−177.454	30	−10.773	12
HEFSVDMTCEGCSNAVTR	−214.700	4	−10.751	13
MQLGGRPPVPR	−191.941	19	−10.686	14
MLEAYNLTEK	−171.044	34	−10.546	15
TEALLGDEQAR	−141.213	43	−10.505	16
CTDLQEEDLK	−134.035	48	−10.478	17
NWWTDSSAEK	−208.414	5	−10.461	18
DGAADWWLGVAGDMFR	−219.080	3	−10.456	19
SIQEIQELDK	−135.654	46	−10.426	20
EAFNMIDQNR	−183.519	24	−10.415	21
DGPPGLPGPPGPPGTPGK	−181.685	27	−10.344	22
VMDSDEDDDY	−138.864	44	−10.329	23
QNPSNPLQR	−181.856	26	−10.277	24
ILESINESALK	−161.908	37	−10.224	25
HRPELIDYGK	−192.496	17	−10.220	26
EVEEDEYK	−134.773	47	−10.194	27
EVFEDAVEIR	−160.843	38	−10.175	28
LHDVDFYK	−186.278	23	−10.169	29
QVIPGLEQSLLDMCVGEK	−200.148	9	−10.127	30
GHYTEGAELVDSVLDVVR	−202.750	7	−9.996	31
ETIPDIDPNAK	−143.443	41	−9.940	32

Peptide sequence	HPEPDOCK rank-1 score	HPEPDOCK rank	Interface-focused Vina score (kcal/mol)	Vina rank
GECPRPNHTAK	−181.587	28	−9.925	33
VTWEPAPGEVK	−196.018	12	−9.836	34
VAPAPARPSGPSK	−202.442	8	−9.812	35
GRPVGFPMR	−188.364	21	−9.801	36
HIDLVEGDEGR	−180.950	29	−9.793	37
AETLNPTPVTAK	−167.282	36	−9.784	38
GSPLEEEGGSLK	−133.815	49	−9.750	39
EAESECEQLAK	−138.365	45	−9.728	40
SPPSPCDAAPFR	−187.236	22	−9.714	41
SSTPVPPPPSLR	−194.873	14	−9.672	42
LSPFMADIR	−189.040	20	−9.659	43
GGSGSGPTIEEVD	−157.649	39	−9.648	44
DVTFSPASIEK	−173.829	32	−9.567	45
LEDELVAQK	−142.433	42	−9.526	46
VDEETENTMR	−145.681	40	−9.416	47
ATGPPVSELITK	−168.764	35	−9.360	48
IQLINNMLDK	−175.535	31	−9.358	49

Note: *Peptides are ordered by their interface-focused Vina ranks. HPEPDOCK scores correspond to the top-ranked global docking model for each peptide. Vina scores correspond to the best-scoring representative pose from the expanded 49-peptide interface-focused docking run. Rankings were calculated separately within each method, with rank 1 indicating the most negative score. Scores from the two methods are not directly comparable and do not represent experimentally measured binding affinities. Spearman's rank correlation between the two rankings was ρ = 0.420.*

Table S2. Starting conformers and representative AutoDock Vina poses retained for the five candidate peptide-RANKL systems. *Part A records the three HPEPDOCK starting conformers per peptide; Part B records the three structurally distinct representative Vina poses retained after pooled ranking.*

Sequence	Conformer	HPEPDOCK rank	Cα RMSD to model 1 (Å)	Seed	Vina poses
GASLQDWDFGK	conf1_model_1	1	0	20260913	20
GASLQDWDFGK	conf2_model_2	2	2.53	20260914	20
GASLQDWDFGK	conf3_model_3	3	2.575	20260915	20
GGYEDPYYGYDDGYAVR	conf1_model_1	1	0	20261013	20
GGYEDPYYGYDDGYAVR	conf2_model_2	2	5.991	20261014	20
GGYEDPYYGYDDGYAVR	conf3_model_3	3	6.11	20261015	20
DGAADWWLGVAGDMFR	conf1_model_1	1	0	20261113	19
DGAADWWLGVAGDMFR	conf2_model_2	2	4.984	20261114	20
DGAADWWLGVAGDMFR	conf3_model_3	3	4.323	20261115	20
HEFSVDMTCEGCSNAVTR	conf1_model_1	1	0	20261213	20
HEFSVDMTCEGCSNAVTR	conf2_model_2	2	6.448	20261214	20
HEFSVDMTCEGCSNAVTR	conf3_model_3	3	4.981	20261215	20
NWWTDSSAEK	conf1_model_1	1	0	20261313	20
NWWTDSSAEK	conf2_model_3	3	3.888	20261314	20
NWWTDSSAEK	conf3_model_4	4	4.364	20261315	20

Table S2, Part B. Representative AutoDock Vina poses.

Sequence	Representative pose	Vina score (kcal/mol)	Source conformer	Source mode	Seed	Cα RMSD to primary (Å)
GASLQDWDFGK	1	-12.07	conf1_model_1	1	20260913	0
GASLQDWDFGK	2	-10.366	conf1_model_1	2	20260913	18.05
GASLQDWDFGK	3	-10.06	conf2_model_2	1	20260914	21.689
GGYEDPYYGYDDGYAVR	1	-11.291	conf3_model_3	1	20261015	0
GGYEDPYYGYDDGYAVR	2	-11.209	conf2_model_2	1	20261014	16.913
GGYEDPYYGYDDGYAVR	3	-11.05	conf3_model_3	2	20261015	6.835
DGAADWWLGVAGDMFR	1	-10.438	conf1_model_1	1	20261113	0
DGAADWWLGVAGDMFR	2	-10.302	conf1_model_1	2	20261113	16.117
DGAADWWLGVAGDMFR	3	-10.007	conf2_model_2	1	20261114	9.901
HEFSVDMTCEGCSNAVTR	1	-10.751	conf2_model_2	1	20261214	0
HEFSVDMTCEGCSNAVTR	2	-10.511	conf2_model_2	2	20261214	6.816
HEFSVDMTCEGCSNAVTR	3	-10.235	conf2_model_2	3	20261214	24.593
NWWTDSSAEK	1	-10.502	conf1_model_1	1	20261313	0
NWWTDSSAEK	2	-10.069	conf3_model_4	1	20261315	24.432
NWWTDSSAEK	3	-9.916	conf1_model_1	2	20261313	17.227

Table S3. Extended AlphaFold 3 interface-confidence metrics for the five predicted peptide-RANKL complexes. *Mean and median interface PAE values complement the minimum interface PAE values reported in the main manuscript. Lower PAE indicates greater confidence in predicted relative residue positions.*

Peptide	Sequence	Length	ipTM	pTM	Ranking score	Mean interface PAE (Å)	Median interface PAE (Å)
P1	GASLQDWDFGK	11	0.52	0.89	0.63	14.1	12.2
P5	NWWTDSSAEK	10	0.44	0.89	0.56	15.6	13.9
P3	DGAADWWLGVAGDMFR	16	0.42	0.86	0.55	16.6	15.3
P2	GGYEDPYYGYDDGYAVR	17	0.33	0.86	0.48	19.2	18.2
P4	HEFSVDMTCEGCSNAVTR	18	0.2	0.83	0.38	22.1	22.4

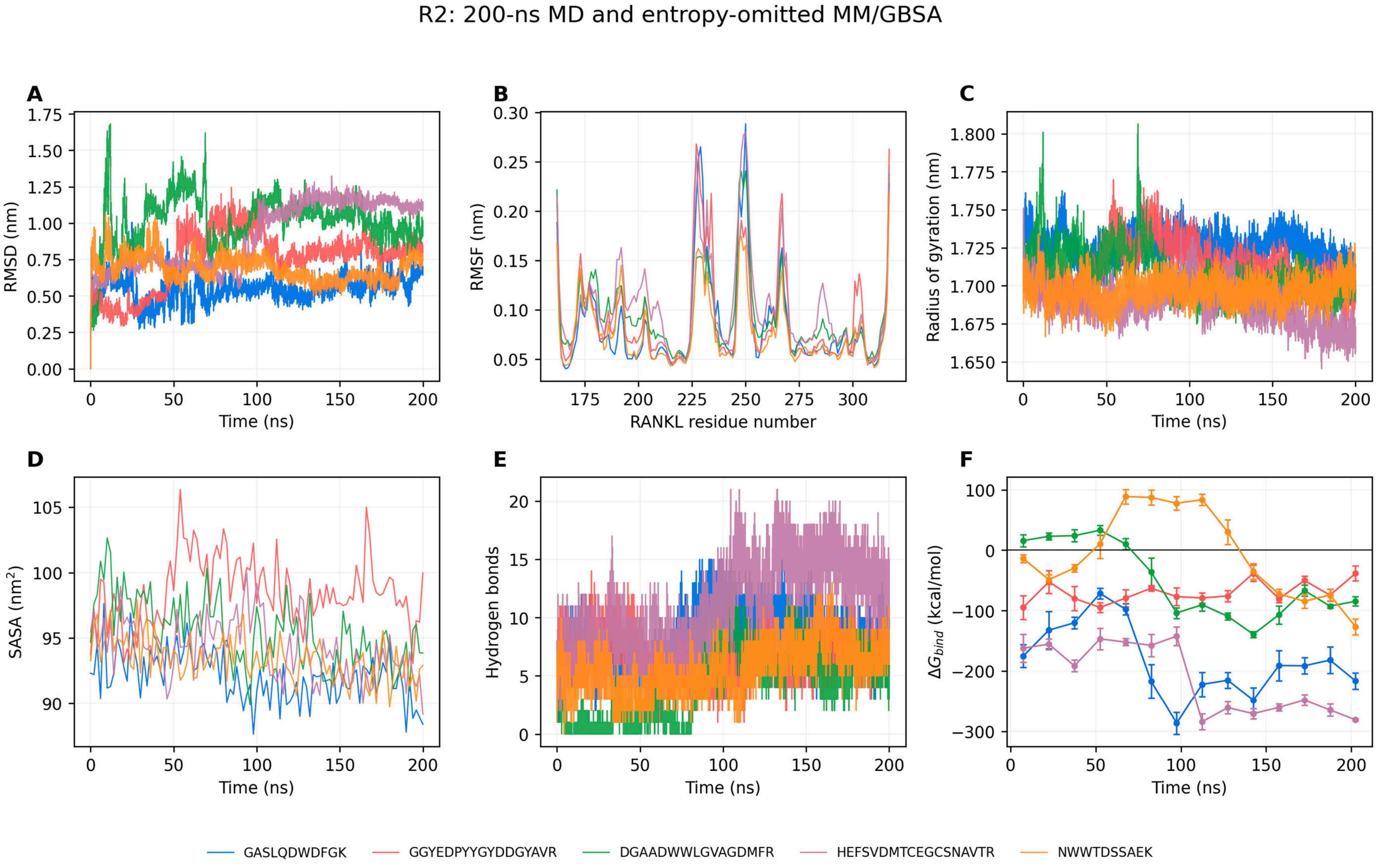

Figure S4. Independent replicate R2 molecular-dynamics and entropy-omitted MM/GBSA profiles for the five peptide-RANKL systems. (A) Peptide RMSD after fitting the RANKL backbone; (B) RANKL-backbone RMSF; (C) radius of gyration of the receptor-peptide protein group; (D) solvent-accessible surface area; (E) peptide-RANKL hydrogen bonds; and (F) time-resolved MM/GBSA endpoint estimates. The panel-F error bars describe variation among correlated snapshots within each time window and are not independent uncertainty estimates.

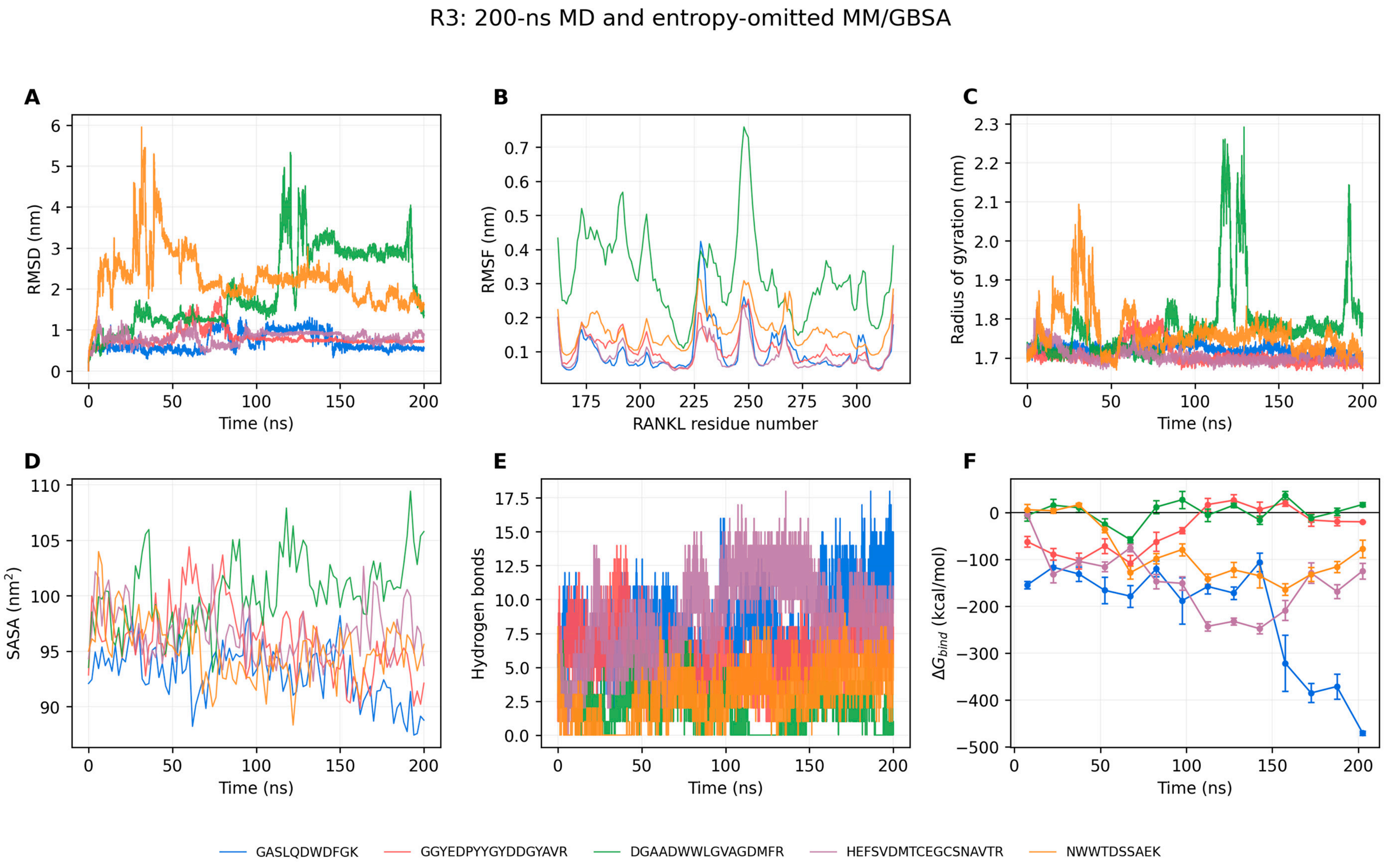

Figure S5. Independent replicate R3 molecular-dynamics and entropy-omitted MM/GBSA profiles for the five peptide-RANKL systems. Panel definitions and statistical interpretation are the same as in Figure S4. The pronounced between-replicate differences, particularly for selected peptides, show that a single trajectory should not be interpreted as a definitive affinity ranking.

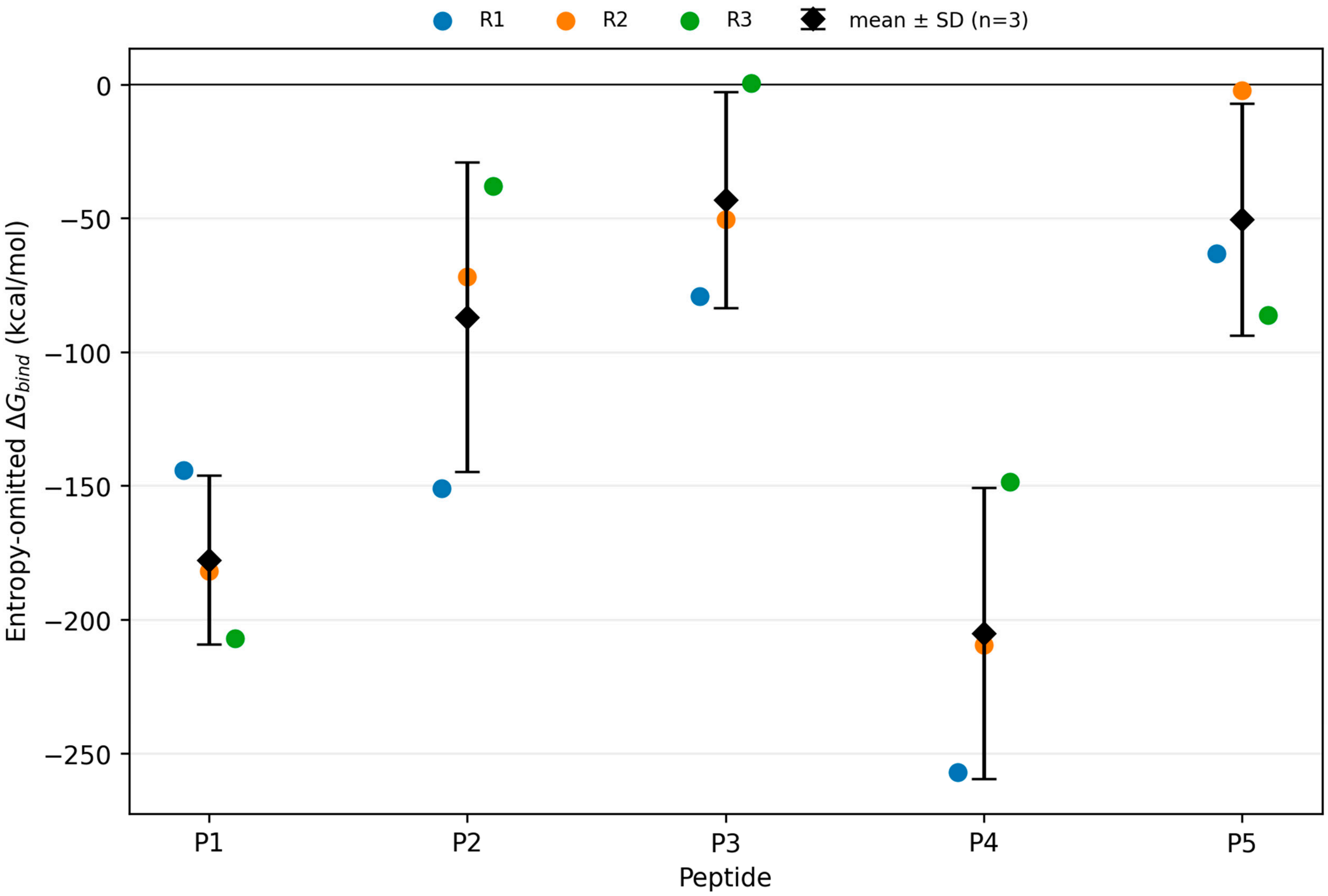

Figure S6. Entropy-omitted MM/GBSA estimates from three independently seeded 200-ns trajectories. Colored points are individual trajectory means; black diamonds and error bars are the across-replicate mean and sample SD (n = 3). Conformational entropy was not included. The estimates are model-dependent and are used only for qualitative prioritization; differences in peptide length and net charge may affect the raw values.

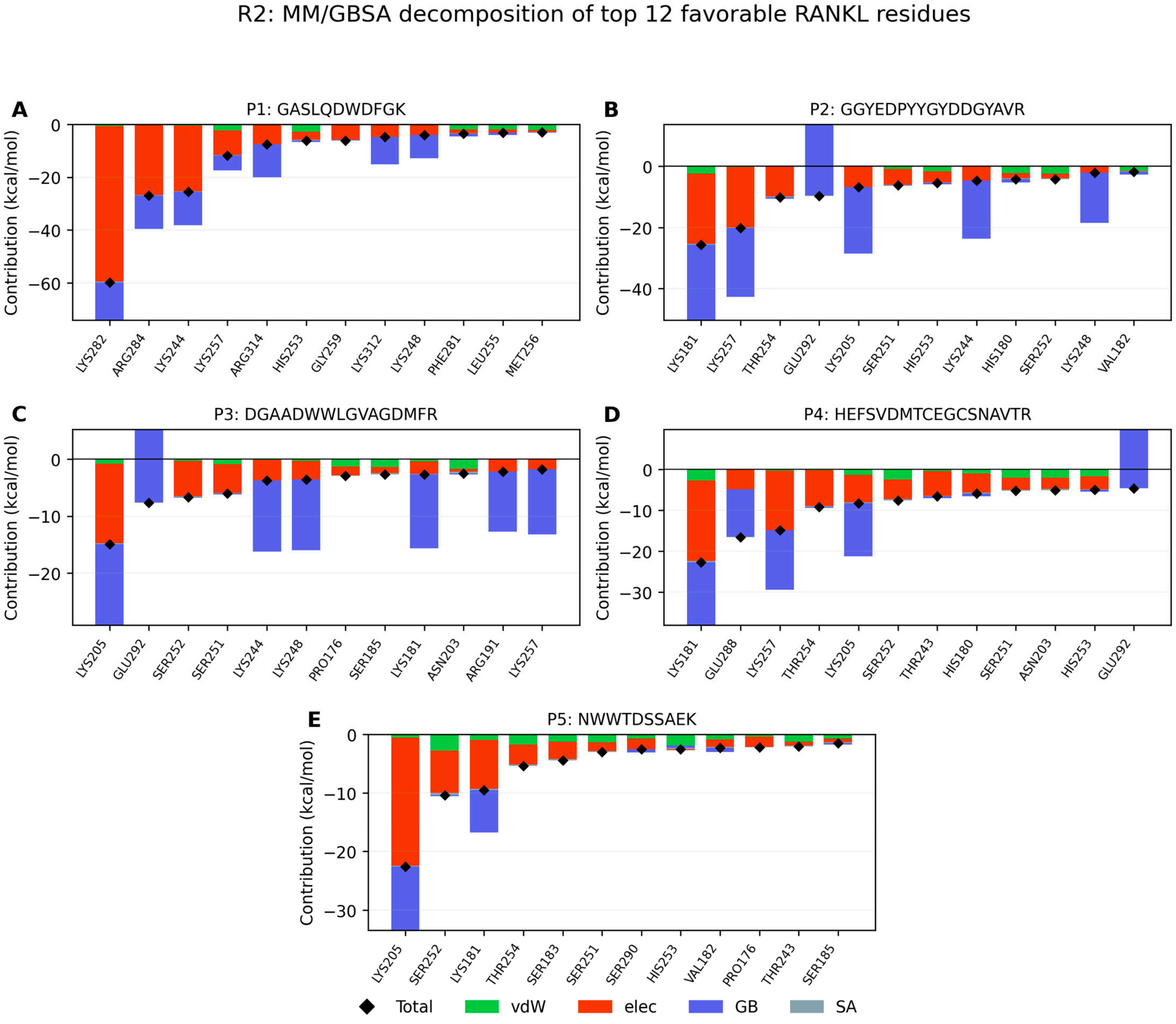

Figure S7. Residue-wise MM/GBSA energy decomposition for the R2 trajectory. Values are descriptive components from one independently seeded trajectory and do not constitute independent residue-level uncertainty estimates or experimentally validated energetic hotspots.

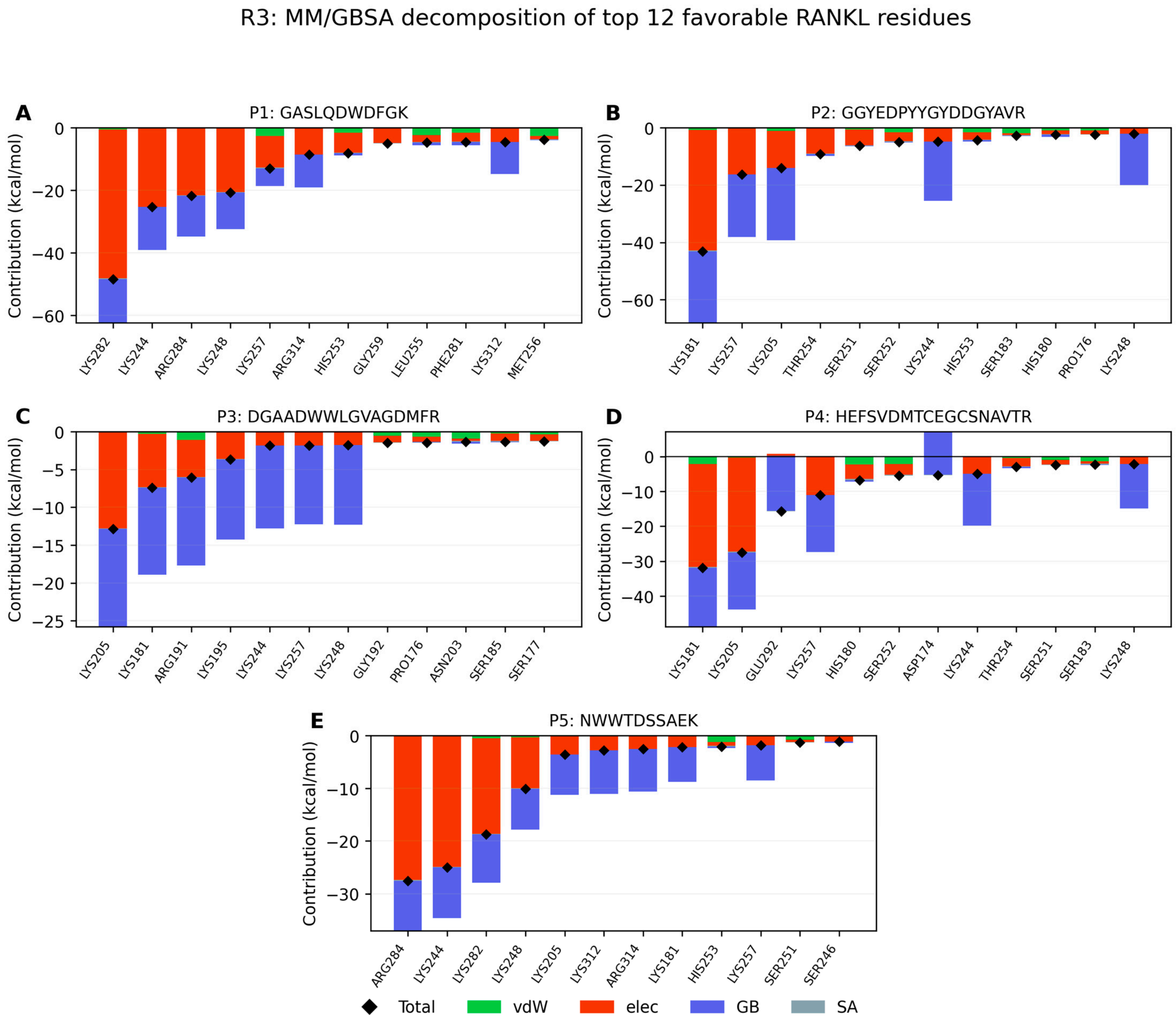

Figure S8. Residue-wise MM/GBSA energy decomposition for the R3 trajectory. Differences from the R1 and R2 profiles reflect conformational and sampling variability and support conservative interpretation of individual residue contributions.

Table S4. Entropy-omitted MM/GBSA estimates summarized over consecutive 15-ns windows for R1-R3. Values are in kcal/mol and are reported as the window mean and within-window SD. Because snapshots within each trajectory are temporally correlated, the within-window SD is descriptive and is not an independent uncertainty estimate. Replicate-level uncertainty is reported in Table S8.

Table S4, Part A. R1 window-resolved estimates.

Window center (ns)	P1 mean	P1 within-window SD	P2 mean	P2 within-window SD	P3 mean	P3 within-window SD	P4 mean	P4 within-window SD	P5 mean	P5 within-window SD
7.5	-173.88	46.06	-97.00	59.05	-51.44	15.97	-64.27	61.92	-38.15	45.96
22.5	-167.45	93.76	-177.10	12.06	-40.15	16.64	-167.57	57.21	-64.41	36.86
37.5	-130.76	24.03	-113.77	53.69	-34.96	15.52	-327.87	91.33	-52.57	25.00
52.5	-121.92	45.88	-116.02	32.51	-44.76	23.41	-279.02	35.78	-67.55	24.54
67.5	-183.48	17.53	-125.88	57.66	-76.64	16.58	-257.60	68.43	-64.08	28.58
82.5	-132.56	56.17	-176.84	35.98	-80.42	16.18	-178.57	39.52	-33.40	44.92
97.5	-151.55	31.98	-155.17	22.29	-40.99	41.21	-244.80	40.98	-4.03	55.96
112.5	-139.15	32.55	-144.27	17.00	-55.69	49.35	-249.90	58.14	-65.66	46.74
127.5	-147.67	38.44	-193.88	16.31	-118.38	22.84	-284.04	42.13	-72.69	36.70
142.5	-146.14	30.50	-170.69	31.89	-107.18	32.30	-305.83	34.38	-86.03	30.55
157.5	-157.19	30.74	-161.84	26.23	-118.68	14.88	-300.44	26.01	-73.77	31.87
172.5	-169.34	33.39	-161.87	22.65	-128.86	11.65	-310.27	41.49	-90.30	22.15
187.5	-88.43	26.77	-155.01	22.80	-105.62	26.79	-340.73	30.94	-95.58	30.24
202.5	-55.35	18.91	-198.55	23.00	-144.75	39.03	-329.02	37.92	-108.50	13.30

Table S4, Part B. R2 window-resolved estimates.

Window center (ns)	P1 mean	P1 within-window SD	P2 mean	P2 within-window SD	P3 mean	P3 within-window SD	P4 mean	P4 within-window SD	P5 mean	P5 within-window SD
7.5	-175.01	53.71	-94.66	55.37	15.84	27.84	-162.08	65.70	-13.90	19.31
22.5	-132.29	81.92	-52.27	49.65	22.98	14.74	-155.48	23.69	-48.71	21.39
37.5	-120.15	26.53	-79.96	57.25	24.08	28.04	-191.43	27.34	-29.60	17.59
52.5	-71.55	23.61	-94.67	21.11	33.08	21.45	-146.77	46.47	10.66	63.37
67.5	-97.67	26.24	-79.40	40.26	10.29	25.25	-152.09	16.31	88.79	33.71
82.5	-216.96	72.89	-62.98	12.75	-36.08	62.35	-157.44	49.10	87.45	31.80
97.5	-286.45	51.08	-76.83	40.77	-103.82	24.81	-142.22	42.72	77.74	31.05
112.5	-222.06	52.73	-79.09	22.01	-90.35	28.15	-284.11	34.10	83.62	24.05
127.5	-215.32	36.38	-75.70	26.07	-109.40	15.88	-261.02	28.70	30.09	57.34
142.5	-248.40	53.93	-38.12	35.54	-139.19	13.33	-270.87	22.53	-35.24	35.04
157.5	-190.89	70.77	-80.54	18.61	-106.86	42.68	-260.56	16.08	-72.33	22.12
172.5	-191.30	36.00	-50.13	20.26	-66.69	24.13	-248.47	23.48	-84.52	30.75
187.5	-181.87	62.33	-74.38	27.89	-92.66	8.84	-264.84	28.11	-74.03	26.87
202.5	-216.40	23.38	-38.45	21.14	-84.43	13.24	-281.11	2.58	-126.71	22.25

Table S4, Part C. R3 window-resolved estimates.

Window center (ns)	P1 mean	P1 within-window SD	P2 mean	P2 within-window SD	P3 mean	P3 within-window SD	P4 mean	P4 within-window SD	P5 mean	P5 within-window SD
7.5	-154.67	22.54	-62.08	31.17	-6.34	33.46	-3.69	24.89	5.90	33.70
22.5	-116.69	41.07	-88.82	32.19	15.56	35.20	-131.42	47.52	3.75	12.34
37.5	-131.19	43.41	-103.27	47.86	10.20	25.83	-104.03	21.73	16.47	10.19
52.5	-165.91	74.10	-71.44	40.91	-24.82	30.33	-115.56	28.47	-35.88	18.08
67.5	-178.56	66.10	-108.11	47.67	-58.25	18.08	-75.09	22.43	-128.28	39.16
82.5	-120.28	44.76	-62.15	53.09	11.66	36.61	-147.13	39.77	-98.20	28.85
97.5	-188.13	139.39	-38.37	19.26	27.23	51.93	-151.11	41.34	-79.07	36.88
112.5	-157.89	41.09	16.94	35.71	-5.59	35.54	-242.79	26.32	-141.54	28.59
127.5	-171.77	36.76	26.13	35.54	15.50	12.94	-232.15	21.43	-122.21	44.61
142.5	-106.27	52.70	6.32	43.63	-15.74	23.68	-247.72	28.29	-134.92	67.70
157.5	-321.43	168.50	20.81	19.04	36.56	25.05	-208.73	60.80	-164.13	34.44
172.5	-384.96	54.10	-16.07	34.38	-12.04	21.42	-129.16	57.15	-131.96	39.17
187.5	-371.20	75.97	-19.25	24.72	1.58	23.08	-168.27	42.28	-115.80	34.43
202.5	-470.35	7.76	-19.87	1.64	17.08	7.25	-124.62	28.91	-77.05	32.47

Table S5. Locally relaxed alanine-substitution energy estimates for all 54 mutable peptide positions. Wild-type and true-alanine mutant topologies were independently minimized in the OBC-II implicit-solvent model using one 150-200-ns structural medoid from each of R1-R3. Values are in kcal/mol. The mean and sample SD use the three independently derived medoids as the statistical unit (n = 3), not individual trajectory frames. Mutant production simulations and conformational entropy were not included; therefore, these values identify qualitative energy-sensitive positions rather than experimentally validated hotspots.

Peptide	Sequence	Mutation	R1	R2	R3	Mean	Across-replicate SD	Minimum	Maximum
P1	GASLQDWDFGK	SER3A	2.39	7.57	11.61	7.19	4.62	2.39	11.61
P1	GASLQDWDFGK	LEU4A	6.53	3.14	3.80	4.49	1.80	3.14	6.53
P1	GASLQDWDFGK	GLN5A	17.98	7.78	7.66	11.14	5.93	7.66	17.98
P1	GASLQDWDFGK	ASP6A	54.28	31.90	75.37	53.85	21.74	31.90	75.37
P1	GASLQDWDFGK	TRP7A	4.84	1.47	1.57	2.63	1.92	1.47	4.84
P1	GASLQDWDFGK	ASP8A	34.27	83.87	100.13	72.76	34.31	34.27	100.13
P1	GASLQDWDFGK	PHE9A	1.70	6.24	1.17	3.03	2.78	1.17	6.24
P1	GASLQDWDFGK	LYS11A	-63.48	87.07	64.74	29.44	81.25	-63.48	87.07
P2	GGYEDPYYGYDDGYAVR	TYR3A	12.41	-1.92	-3.67	2.27	8.82	-3.67	12.41
P2	GGYEDPYYGYDDGYAVR	GLU4A	46.38	74.55	58.68	59.87	14.12	46.38	74.55
P2	GGYEDPYYGYDDGYAVR	ASP5A	54.34	77.38	58.32	63.35	12.32	54.34	77.38
P2	GGYEDPYYGYDDGYAVR	TYR7A	7.45	9.66	14.98	10.70	3.87	7.45	14.98
P2	GGYEDPYYGYDDGYAVR	TYR8A	0.17	-1.15	4.66	1.23	3.05	-1.15	4.66
P2	GGYEDPYYGYDDGYAVR	TYR10A	0.51	-20.33	11.29	-2.84	16.07	-20.33	11.29
P2	GGYEDPYYGYDDGYAVR	ASP11A	67.97	8.04	-37.99	12.67	53.13	-37.99	67.97
P2	GGYEDPYYGYDDGYAVR	ASP12A	49.82	-21.32	-2.75	8.58	36.90	-21.32	49.82
P2	GGYEDPYYGYDDGYAVR	TYR14A	2.38	3.06	1.54	2.33	0.76	1.54	3.06
P2	GGYEDPYYGYDDGYAVR	VAL16A	-0.43	-4.76	0.46	-1.58	2.79	-4.76	0.46
P2	GGYEDPYYGYDDGYAVR	ARG17A	-34.31	3.76	-16.98	-15.84	19.06	-34.31	3.76
P3	DGAADWWLGVAGDMFR	ASP1A	6.73	-46.93	-76.62	-38.94	42.25	-76.62	6.73
P3	DGAADWWLGVAGDMFR	ASP5A	11.99	-36.98	-44.69	-23.23	30.74	-44.69	11.99
P3	DGAADWWLGVAGDMFR	TRP6A	12.12	-0.15	-0.13	3.95	7.08	-0.15	12.12
P3	DGAADWWLGVAGDMFR	TRP7A	17.04	20.20	1.54	12.93	9.99	1.54	20.20
P3	DGAADWWLGVAGDMFR	LEU8A	1.22	0.09	4.42	1.91	2.24	0.09	4.42
P3	DGAADWWLGVAGDMFR	VAL10A	-0.57	5.73	-0.37	1.60	3.58	-0.57	5.73
P3	DGAADWWLGVAGDMFR	ASP13A	74.10	-6.86	-11.25	18.66	48.06	-11.25	74.10
P3	DGAADWWLGVAGDMFR	MET14A	1.46	-0.89	0.70	0.42	1.20	-0.89	1.46
P3	DGAADWWLGVAGDMFR	PHE15A	2.45	3.84	-0.24	2.02	2.08	-0.24	3.84
P3	DGAADWWLGVAGDMFR	ARG16A	-12.03	23.09	5.38	5.48	17.56	-12.03	23.09
P4	HEFSVDMTCEGCSNAVTR	HIS1A	24.10	13.92	-3.60	11.48	14.01	-3.60	24.10
P4	HEFSVDMTCEGCSNAVTR	GLU2A	12.42	-51.23	-23.24	-20.68	31.90	-51.23	12.42
P4	HEFSVDMTCEGCSNAVTR	PHE3A	4.13	7.34	-1.68	3.26	4.57	-1.68	7.34
P4	HEFSVDMTCEGCSNAVTR	SER4A	3.78	4.84	-6.72	0.64	6.39	-6.72	4.84
P4	HEFSVDMTCEGCSNAVTR	VAL5A	3.43	2.54	-8.63	-0.89	6.72	-8.63	3.43

Peptide	Sequence	Mutation	R1	R2	R3	Mean	Across-replicate SD	Minimum	Maximum
P4	HEFSVDMTCEGCSNAVTR	ASP6A	86.07	-22.96	27.73	30.28	54.56	-22.96	86.07
P4	HEFSVDMTCEGCSNAVTR	MET7A	3.87	0.90	-0.14	1.54	2.08	-0.14	3.87
P4	HEFSVDMTCEGCSNAVTR	THR8A	11.12	28.02	40.78	26.64	14.88	11.12	40.78
P4	HEFSVDMTCEGCSNAVTR	CYS9A	1.77	2.33	4.06	2.72	1.19	1.77	4.06
P4	HEFSVDMTCEGCSNAVTR	GLU10A	134.60	102.08	52.03	96.24	41.59	52.03	134.60
P4	HEFSVDMTCEGCSNAVTR	CYS12A	-15.86	3.93	-2.91	-4.95	10.05	-15.86	3.93
P4	HEFSVDMTCEGCSNAVTR	SER13A	17.29	1.34	0.63	6.42	9.42	0.63	17.29
P4	HEFSVDMTCEGCSNAVTR	ASN14A	8.03	1.68	-0.74	2.99	4.53	-0.74	8.03
P4	HEFSVDMTCEGCSNAVTR	VAL16A	3.93	0.91	8.04	4.30	3.58	0.91	8.04
P4	HEFSVDMTCEGCSNAVTR	THR17A	0.43	0.41	0.51	0.45	0.06	0.41	0.51
P4	HEFSVDMTCEGCSNAVTR	ARG18A	49.05	97.38	6.77	51.07	45.34	6.77	97.38
P5	NWWTDSSAEK	ASN1A	-2.52	-0.03	-3.65	-2.07	1.85	-3.65	-0.03
P5	NWWTDSSAEK	TRP2A	0.37	-0.20	7.71	2.63	4.41	-0.20	7.71
P5	NWWTDSSAEK	TRP3A	9.06	23.89	-8.40	8.18	16.16	-8.40	23.89
P5	NWWTDSSAEK	THR4A	4.36	-2.72	-0.81	0.28	3.66	-2.72	4.36
P5	NWWTDSSAEK	ASP5A	29.77	33.43	32.72	31.98	1.94	29.77	33.43
P5	NWWTDSSAEK	SER6A	-1.11	25.19	1.43	8.51	14.51	-1.11	25.19
P5	NWWTDSSAEK	SER7A	-0.52	-0.66	-1.07	-0.75	0.29	-1.07	-0.52
P5	NWWTDSSAEK	GLU9A	50.57	-14.47	107.03	47.71	60.80	-14.47	107.03
P5	NWWTDSSAEK	LYS10A	-8.81	22.46	-37.88	-8.08	30.18	-37.88	22.46

Table S6. Molecular-dynamics protocol and replicate design. All five peptide systems were simulated for three independently seeded 200-ns trajectories using identical production settings.

Parameter	Value
Independent unit	One independently seeded 200-ns trajectory; n = 3 per peptide
Systems	Five peptide-RANKL complexes; 15 production trajectories in total
Starting structures	R1-R3 used the same selected starting complex for each peptide; initial velocities differed
Integrator and time step	Molecular dynamics; 2 fs
Production length	100,000,000 steps; 200 ns per trajectory
Temperature coupling	V-rescale; 298 K; tau-t = 0.1 ps
Pressure coupling	C-rescale; 1 bar; tau-p = 2.0 ps
Electrostatics and cutoffs	Particle-mesh Ewald electrostatics; Verlet cutoff scheme
Constraints	Hydrogen-bond constraints with LINCS
MM/GBSA sampling	101 snapshots per trajectory at 2-ns intervals
MM/GBSA model	Single-trajectory OBC-II/SASA endpoint model; conformational entropy omitted
Uncertainty	Sample SD across the three trajectory means; framewise SD is descriptive only

Table S6, Part B. Random seeds used to generate initial velocities.

Peptide	R1 gen-seed	R1 ld-seed	R2 gen-seed	R2 ld-seed	R3 gen-seed	R3 ld-seed
P1	202609161	202610161	202609261	202610261	202609361	202610361
P2	202609162	202610162	202609262	202610262	202609362	202610362
P3	202609163	202610163	202609263	202610263	202609363	202610363
P4	202609164	202610164	202609264	202610264	202609364	202610364
P5	202609165	202610165	202609265	202610265	202609365	202610365

Table S7. Across-replicate summary of molecular-dynamics observables. Means and sample SDs were calculated from the three trajectory means (n = 3), both over the complete 0-200-ns trajectories and over the final 150-200-ns interval. RMSD denotes peptide RMSD after fitting the RANKL backbone; Rg and SASA refer to the receptor-peptide protein group; hydrogen bonds were calculated between RANKL residues 162-317 and the peptide.

Peptide	Metric	Unit	Mean, 0-200 ns	SD, 0-200 ns	Mean, 150-200 ns	SD, 150-200 ns
P1	RMSD	nm	0.582	0.103	0.567	0.042
P1	Rg	nm	1.716	0.012	1.709	0.013
P1	SASA	nm^2	91.850	1.266	90.945	0.384
P1	H-bonds	count	8.143	0.161	8.779	1.396
P2	RMSD	nm	0.964	0.319	1.020	0.436
P2	Rg	nm	1.717	0.014	1.711	0.028
P2	SASA	nm^2	97.962	1.710	97.260	3.852
P2	H-bonds	count	5.990	0.427	6.127	1.023
P3	RMSD	nm	1.500	0.489	1.918	0.889
P3	Rg	nm	1.751	0.039	1.758	0.052
P3	SASA	nm^2	97.612	2.454	97.148	4.179
P3	H-bonds	count	3.373	1.173	3.864	1.818
P4	RMSD	nm	1.155	0.511	1.332	0.644
P4	Rg	nm	1.711	0.026	1.704	0.031
P4	SASA	nm^2	95.787	1.358	94.518	2.366
P4	H-bonds	count	9.864	1.363	11.924	2.940
P5	RMSD	nm	1.468	0.781	1.235	0.568
P5	Rg	nm	1.720	0.037	1.707	0.022
P5	SASA	nm^2	94.199	0.822	93.238	1.512
P5	H-bonds	count	4.112	1.456	5.947	1.595

Table S8. Full entropy-omitted MM/GBSA summary across three independent trajectories. Each R1-R3 value is the mean of 101 snapshots sampled every 2 ns. The reported uncertainty is the sample SD of the three trajectory means, not framewise SEM. Ranking changed among replicates, and the values should be interpreted as qualitative endpoint estimates rather than evidence that one peptide has definitively stronger affinity.

Peptide	Sequence	Length	R1 mean	R2 mean	R3 mean	Across-replicate mean	Sample SD	Ranks R1/R2/R3
P1	GASLQDWDFGK	11	-144.22	-181.84	-207.04	-177.70	31.61	3/2/1
P2	GGYEDPYYGYDDGYAVR	17	-150.93	-71.76	-38.01	-86.90	57.96	2/3/4
P3	DGAADWWLGVAGDMFR	16	-79.28	-50.37	0.46	-43.06	40.37	4/4/5
P4	HEFSVDMTCEGCSNAVTR	18	-257.26	-209.50	-148.66	-205.14	54.43	1/1/2
P5	NWWTDSSAEK	10	-63.21	-2.31	-86.13	-50.55	43.32	5/5/3
